# Is Autophagy Targeting a Valid Adjuvant Strategy in Conjunction with Tyrosine Kinase Inhibitors?

**DOI:** 10.3390/cancers16172989

**Published:** 2024-08-28

**Authors:** Ahmed M. Elshazly, Jingwen Xu, Nebras Melhem, Alsayed Abdulnaby, Aya A. Elzahed, Tareq Saleh, David A. Gewirtz

**Affiliations:** 1 Department of Pharmacology and Toxicology, School of Medicine, Virginia Commonwealth University, 401 College St., Richmond, VA 23298, USA; elshazlyam@vcu.edu; 2 Department of Pharmacology and Toxicology, Faculty of Pharmacy, Kafrelsheikh University, Kafrelsheikh 33516, Egypt; aaya9247@gmail.com; 3 School of Pharmacy, Guangdong Pharmaceutical University, Guangzhou 510006, China; jingwen_xu@gdpu.edu.cn; 4 Department of Anatomy, Physiology and Biochemistry, Faculty of Medicine, The Hashemite University, Zarqa 13133, Jordan; nebras@hu.edu.jo; 5 Department of Pharmacognosy, Faculty of Pharmacy, Kafrelsheikh University, Kafrelsheikh 33516, Egypt; alsayed.allam@pharm.kfs.edu.eg; 6 Department of Pharmacology and Public Health, Faculty of Medicine, Hashemite University, Zarqa 13133, Jordan; tareq@hu.edu.jo

**Keywords:** autophagy, cytoprotective, cytotoxic, tyrosine kinase, resistance

## Abstract

**Simple Summary:**

Tyrosine kinase inhibitors (TKIs) have demonstrated effectiveness in a variety of malignancies. As is the case for many different classes of drugs, tyrosine kinase inhibitors induce autophagy in various tumor cell models. Autophagy is a cellular degradative machinery that can be protective or cytotoxic to the cells, and in some cases, autophagy has no detectable influence on cell sensitivity to chemotherapy. This review demonstrates that cytoprotective and cytotoxic autophagy are the main forms induced by TKIs and that targeting or modulating autophagy can potentially enhance the tumor cell response to tyrosine kinase inhibitors.

**Abstract:**

Tyrosine kinase inhibitors (TKIs) represent a relatively large class of small-molecule inhibitors that compete with ATP for the catalytic binding site of tyrosine kinase proteins. While TKIs have demonstrated effectiveness in the treatment of multiple malignancies, including chronic myelogenous leukemia, gastrointestinal tumors, non-small cell lung cancers, and HER2-overexpressing breast cancers, as is almost always the case with anti-neoplastic agents, the development of resistance often imposes a limit on drug efficacy. One common survival response utilized by tumor cells to ensure their survival in response to different stressors, including anti-neoplastic drugs, is that of autophagy. The autophagic machinery in response to TKIs in multiple tumor models has largely been shown to be cytoprotective in nature, although there are a number of cases where autophagy has demonstrated a cytotoxic function. In this review, we provide an overview of the literature examining the role that autophagy plays in response to TKIs in different preclinical tumor model systems in an effort to determine whether autophagy suppression or modulation could be an effective adjuvant strategy to increase efficiency and/or overcome resistance to TKIs.

## 1. Introduction

**Figure 1:**
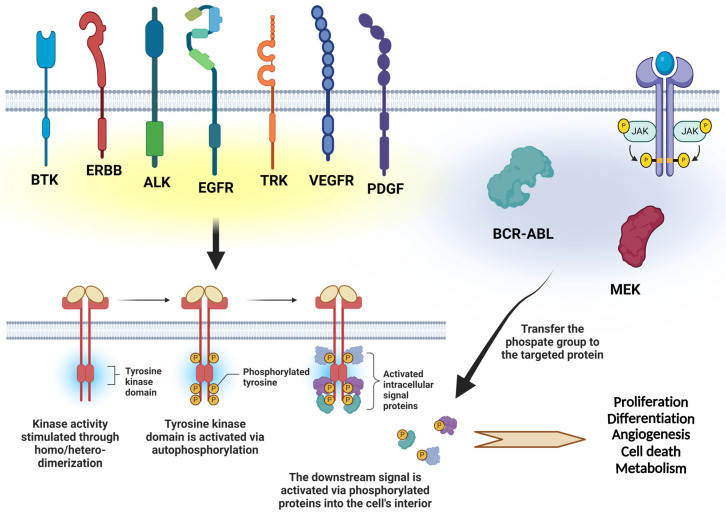
Tyrosine kinase receptors and their role in cancer. The different tyrosine kinase receptors, including epidermal growth factor receptors (ERBB and EGFR), anaplastic lymphoma kinase (ALK), Bruton’s tyrosine kinase (BTK), tropomyosin receptor kinase (TRK), platelet-derived growth factor (PDGF), and vascular endothelial growth factor receptor (VEGFR), are activated via homo- or hetero-dimerization. This activation leads to the autophosphorylation of the kinase domain, which then transfers the phosphate group to the targeted proteins. BCR–ABL, Janus kinase (JAK), and mitogen-activated protein kinase (MEK) catalyze the phosphorylation of various target proteins upon their activation. Ultimately, tyrosine kinase activities result in the activation of various signaling pathways involved in cellular proliferation, differentiation, angiogenesis, metabolism, and cell death.

Tyrosine kinases are a family of proteins that catalyze the transfer of a phosphate group from ATP to tyrosine residues on target proteins, transducing various forms of intracellular signals. Tyrosine kinases are involved in the regulation of cell growth, differentiation, and cell death [1,2]. The dysregulation of tyrosine kinase expression/activity is linked to a wide range of disorders, primarily carcinogenesis and tumor development [2]. Several studies have revealed that multiple oncogenes exhibit tyrosine kinase activity, contributing to the uncontrolled proliferation of transformed cells or cells in the process of transformation [3]. Furthermore, the dysregulated expression of tyrosine kinases is linked to various characteristics of malignant cells, including invasion, neovascularization, metastasis, and resistance to chemotherapy [2,4]. Therefore, the targeting of tyrosine kinases has emerged as a promising antineoplastic therapeutic strategy. Consequently, small-molecule inhibitors that target tyrosine kinases have been developed and have gained approval for clinical use. Two different modes of action for TKIs exist. The ATP-competitive inhibitors compete with intracellular ATP for phosphorylation of the catalytic site of tyrosine kinases, such as gefitinib, pazopanib, ruxolitinib, and vandetanib [5,6]. The non-ATP competitive inhibitors act by inducing a conformational shift in the target kinase, which is no longer able to function, such as imatinib, sorafenib, axitinib, and nilotinib [6,7]. These agents target a wide range of tyrosine kinase proteins, including epidermal growth factor receptors (ERBB and EGFR), Bruton’s tyrosine kinase (BTK), anaplastic lymphoma kinase (ALK), tropomyosin receptor kinase (TRK), Janus kinase (JAK), vascular endothelial growth factor receptor (VEGFR), platelet-derived growth factor (PDGF), BCR–ABL, and mitogen-activated protein kinase (MEK) (Figure 1) [8,9]. Despite their considerable clinical efficacy, many of these agents have been associated with the development of resistance, which has constrained their utility.

Autophagy is a metabolic machinery that evolved primarily to carry out recycling of damaged organellar and suborganellar structures, and thereby to maintain energetic homeostasis [10,11]. Autophagy is largely viewed as a cell survival response that is triggered as a consequence of various stressors, including nutritional deprivation, oxidative stress, and endoplasmic reticulum (ER) stress, mainly elicited by protein misfolding [10,11]. The autophagic process is comprised of a number of interim steps that are initiated by phagophore nucleation and autophagosome formation, followed by lysosomal fusion with the autophagosomes (autophagolysosomes), and finally, cargo degradation and recycling [10,11]. These mechanistic steps, regulated by a complex network of autophagy-related genes (ATG) [10,11], were discussed in detail in previous manuscripts in this series [10,12].

Four different functions of autophagy have been identified, most prominently the resistance-associated cytoprotective form, but also the cell death-associated forms: cytotoxic, the less well-characterized cytostatic, and non-protective forms [13,14]. Autophagy clearly serves as a pro-survival mechanism that mediates the cellular response to stressors such as starvation, nutrient deprivation, hypoxia, organelle/DNA damage, ER stress, and infection [15]. Tumor cells have been shown to utilize autophagy to bolster their proliferation capacity upon stress exposure and thus seem to also depend on cytoprotective autophagy for their survival [16]. The induction of cytoprotective autophagy can be achieved through several pathways, including mTOR inhibition and AMPK activation [17]. Typically, autophagy would exert its cytoprotective function by switching off key apoptotic pathways through, for example, the systematic degradation of pro-apoptotic proteins such as caspase-8 [18]. Alternatively, autophagy would facilitate the inhibition of these proteins, such as the association between UV radiation resistance-associated gene protein (UVRAG), which, through its association with Bax, inhibits its activity as well as its translocation to the mitochondria [19]. On the other hand, autophagy was also found to be capable of exerting a cytotoxic function, wherein induction of autophagy would promote cellular demise. In this form, the cells accumulate both autophagosomes and autolysosomes in the cytoplasm [20]. Furthermore, autophagy often exerts its cytotoxicity through the facilitation of other cell death forms, including necroptosis and apoptosis. For example, autophagy mediates apoptosis through degrading Fap-1, a negative regulator of Fas, where p62 recognizes and binds to Fap-1, leading to its degradation [21]. This, in turn, increases the apoptotic response through FAS phosphorylation. Moreover, in other systems, the autophagy regulatory protein ATG12 binds to the antiapoptotic regulators Bcl-2 and Mcl-1, leading to BAX activation and the immediate triggering of cell death [22]. Additionally, another autophagy regulatory protein, ATG5, can be degraded by calpain to a truncated form, which is in turn translocated to the mitochondria, blocking Bcl-2 and Mcl-1 as well as altering the mitochondrial membrane permeability [23]. Autophagy can also serve as a platform for the assembly of the necroptosis complex, where ATG5 helps in the assembly of the necrosis complex and consists of receptor-interacting protein kinase 1 (RIPK1), receptor-interacting protein kinase 3 (RIPK3), and Fas-associating protein with a death domain (FADD), promoting cell lysis [23]. In contrast to the previous forms, the cytostatic form of autophagy is not fully understood; however, our research group showed that this form of autophagy usually occurs in parallel with senescence and leads to growth arrest [24,25]. The same unclear understanding also applies to non-protective autophagy, where the inhibition or promotion of autophagy does not culminate in a significant change in cell viability [26]. Collectively, these forms are dependent on the cell/tumor type, the stage of the tumor, and the chemical nature of the compound being utilized. 

**Figure 2:**
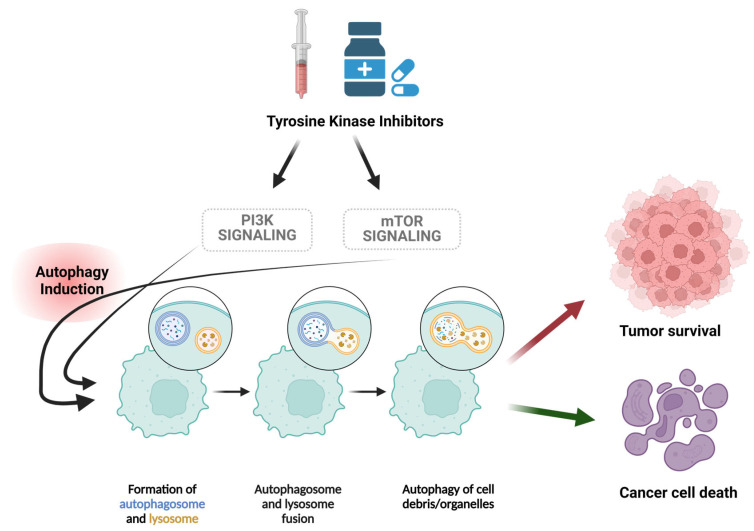
Tyrosine kinase inhibitors induce the autophagic machinery. Tyrosine kinase inhibitors (TKIs) are able to induce the autophagic response in various tumor models through different signaling pathways, including PI3K as well as mTOR pathways. Once autophagy is activated, autophagosomes are formed, which are then fused with lysosomes, forming autophagolysosomes. The latter structure is where degradation of the subcellular organelles takes place. The autophagic response to TKI treatment leads to either cell death (cytotoxic autophagy) or tumor progression (cytoprotective autophagy).

It is within this framework that this review was developed to evaluate the literature assessing autophagy induced by tyrosine kinase inhibitors in various tumor models (Figure 2), as part of a series [10,12,13,27,28,29,30,31,32,33] that explores the role(s) of autophagy in dictating the response to diverse anticancer therapies, in an effort to determine whether autophagy inhibition or modulation could be an effective adjuvant strategy to overcome resistance and increase the efficacy of the clinically approved tyrosine kinase inhibitors.

## 2. Tyrosine Kinase Inhibitors and Autophagy

Focusing on the cytoprotective form of autophagy, preclinical research has shown the possible contribution of autophagy to antitumor drug resistance and considered the modulation or targeting of autophagy as a strategy to increase the effectiveness of chemotherapeutic agents [34,35,36,37]. Furthermore, multiple clinical trials have been initiated targeting the autophagic machinery using chloroquine (CQ) and hydroxychloroquine (HCQ), but with wide variability in their respective outcomes [29,30]. Beside the induction of senescence [38] and ferroptosis [39], tyrosine kinase inhibitors induce autophagy [40]. Tyrosine kinase inhibitors can induce autophagy through various mechanisms, including the reduction of mTOR phosphorylation via Met [41,42], PI3K/AKT, MAPK, and JAK/STAT signaling pathways [43]. Apart from mTOR inhibition, autophagy induction via AMPK activation has also been reported, where the BCR-ABL TKI, nilotinib, activates AMPK, which in turn phosphorylates Unc-51-like autophagy activating kinase 1 (ULK1) [43]. ULK1 activates downstream molecules such as ATG13, ultimately increasing autophagic flux [43].

### 2.1. ErbB Inhbitors

#### 2.1.1. Afatinib

Afatinib is an irreversible ErbB family TKI, approved for treating metastatic non-small cell lung cancer (NSCLC) with an L858R EGFR mutation [44]. Afatinib and its ability to induce autophagy have been investigated in various tumor models. For example, Liu et al. [45] showed that afatinib treatment induced autophagy in the FaDu, HN6, and CAL-27 head and neck squamous cell carcinoma (HNSCC) cell lines, as evidenced by LC3II accumulation and foci formation, p62/SQSTM1 degradation, and increased Beclin 1 expression [45]. Upon autophagy inhibition by the pharmacological agents 3-methyladenine (3-MA) or CQ in combination with afatinib, a pronounced elevation in apoptosis levels over that induced by afitinib alone was reported. Additionally, knockdown of the autophagy regulatory gene, ATG-5, via siRNA, when combined with afatinib, enhanced the cleavage of caspase-3 and PARP, indicative of apoptosis, collectively confirming the cytoprotective role of the autophagy induced by afatinib in HNSCC [45].

In another tumor model, Hu et al. investigated the effect of autophagy inhibition in afatinib-treated H1650 and H1975 lung adenocarcinoma cell lines that harbor activating EGFR mutations [46]. Afatinib induced a robust autophagic response in these cell lines, as evidenced by autophagosome accumulation visualized by transmission electron microscopy (TEM), Cyto-ID dye, which stains the autophagic compartments [47], as well as LC3I/II conversion. While afatinib alone induced growth inhibition and apoptosis, as confirmed by an MTS assay and cleaved PARP levels, autophagy inhibition by CQ or 3-MA significantly increased afatinib-induced cell death, again indicative of a cytoprotective function of autophagy [46]. These outcomes were confirmed in vivo using nude mice inoculated with H1975 cells; here, CQ in combination with afatinib resulted in a more pronounced reduction in tumor volume than each drug alone. Mechanistically, the protein kinase B (AKT)/mammalian target of rapamycin (mTOR) and ERK signaling pathways were found to play key roles in mediating afatinib-induced cytoprotective autophagy [46].

Collectively, these results appear to establish a cytoprotective role of autophagy induced by afatinib in both lung adenocarcinoma and head and neck squamous cell carcinoma, suggesting that autophagy may represent a valid target for enhancing the effectiveness of afatinib-based therapy in these tumor types.

#### 2.1.2. Lapatinib

Lapatinib is a dual tyrosine kinase inhibitor, targeting both HER1 and HER2 receptors, as mentioned in our previous publication [33]. Lapatinib is approved for treating advanced hormone receptor- and HER2-positive breast cancer patients [48]. A number of studies have investigated the role of autophagy as a major stress response to lapatinib in tumor cells. For example, Chen et al. investigated the possible contribution of the autophagic flux to lapatinib resistance in HER2 overexpressing breast cancer cell variants, the parental BT-474 and AU-565 cell lines, and the resistant cells BT-474^LapR^ and AU-565^LapR^ [49]. The resistant cell lines were shown to have elevated baseline levels of autophagy as compared with the parental cells, as evidenced by autophagosome accumulation visualized by TEM and GFP-LC3 puncta formation. Upon combining lapatinib with the autophagy inhibitors CQ, 3-MA, or bafilomycin A1 (BAF A1), significantly increased inhibition of the resistant cell proliferation was noted in comparison with each therapy alone. These results were confirmed by assessment of clonogenic survival, where the combination treatments abrogated colony formation for the resistant cell lines and increased the apoptotic cell population over that for each therapy alone, consistent with the cytoprotective role of lapatinib-induced autophagy and its critical role in mediating resistance. Again, mechanistic studies revealed a central role for the AMPK/mTOR signaling pathway in mediating the protective function of autophagy in this system.

Zhu et al. studied lapatinib using the same BT474 and AU565 cell lines [50]. Lapatinib inhibited the growth of both cell lines and triggered cell death, as demonstrated in a clonogenic survival assay, and promoted apoptosis based on the cleavage of caspase 3 and PARP. Importantly, lapatinib treatment induced autophagy, as confirmed by acridine orange staining, TEM-mediated visualization of autophagosome accumulation, GFP-LC3 puncta formation, and increased LC3 lipidation. Unexpectedly, inhibition of lapatinib-induced autophagy by 3-MA increased rather than inhibiting the proliferative abilities of both cell lines. Additionally, 3-MA protected tumor cells from lapatinib-induced apoptosis, suggesting, in contrast to the findings by Chen et al. [49], a cytotoxic role for the autophagy induced by lapatinib in these cell lines. 

In myeloblastic leukemia, Chen et al. investigated lapatinib and its possible relation to the autophagic machinery [51]. Lapatinib reduced the viability of AML-derived U937 cells using an MTS assay and trypan blue exclusion. Lapatinib induced low levels of apoptosis in U937 cells despite a high degree of cell killing, likely indicative of a different form of cell death. The minimal involvement of apoptosis was confirmed using the caspase inhibitor Z-VAD-fmk, which could not protect most U937 cells from lapatinib toxicity. Lapatinib treatment did promote autophagy, as evidenced by GFP-LC3 puncta formation and acridine orange staining. Upon autophagy inhibition by 3-MA, lapatinib-induced cell death was abrogated, again indicative of the cytotoxic role of autophagy in this system. The putative cytotoxic role of autophagy in these experimental models was further confirmed by studies where autophagy was genetically inhibited via ATG7, ATG5, and Beclin 1-targeting shRNA, where autophagy blockade dramatically reduced sensitivity to Lapatinib. Similar results were also demonstrated with the K562 cell line.

Similar to the previously discussed findings in myeloblastic leukemia [5], Chen et al. [52] showed that lapatinib also induced a cytotoxic form of autophagy in hepatocellular carcinoma (HCC). Lapatinib reduced the viability of several HCC cell lines, including Huh7, HepG2, and HA22T cells. Similar to the findings in myeloblastic leukemia, lapatinib induced significant cell death with a low percentage of sub-G1 cells, indicative of minimal apoptosis, in Huh7, HepG2, and HA22T cells. Lapatinib treatment was again shown to promote autophagy, as confirmed by acridine orange staining, punctuate LC3 aggregation, upregulation of Beclin 1 (ATG6), ATG5, ATG7, and BNIP, and p62/SQSTM1 degradation. Treatment with the autophagy inhibitors 3-MA or CQ effectively blocked lapatinib-mediated cytotoxicity in all three cell lines. The cytotoxic role of autophagy was further confirmed by the genetic inhibition of autophagy using ATG5, ATG7, and Beclin 1-targeting shRNA, where the autophagy inhibition rescued the HCC cells from lapatinib-mediated cell death.

Janser et al. also studied lapatinib, but in esophageal carcinoma using the HER2-positive OE19 cell line [53]. Lapatinib treatment induced autophagic flux in both cell lines, as evidenced by LC3I/II conversion as well as via LC3 puncta formation. Basal autophagy levels were higher in lapatinib-resistant OE19 cells in comparison with their sensitive counterparts. Moreover, lapatinib treatment further increased the levels of autophagy in the resistant cells. Upon treatment with the early-stage autophagy inhibitor, VPS34-IN1 [54], or CQ in combination with lapatinib, a further reduction in the viability of parental cells compared with each treatment alone was observed, indicative of cytoprotective autophagy. Intriguingly, autophagy inhibition alone or in combination with lapatinib showed a significant, but almost similar, sensitization in the resistant cell lines. However, in a clonogenic assay, the resistant cells only responded to the combination of lapatinib with VPS34-IN1, the early-stage autophagy inhibitor [54]. Collectively, these results highlight the cytoprotective role of autophagy mediated by lapatinib in esophageal carcinoma and its possible contribution to lapatinib resistance.

Kang et al. [55] investigated autophagy inhibition in bladder cancer in combination with lapatinib and another EGFR inhibitor, gefitinib. While both EGFR inhibitors were mildly effective in interfering with the growth of T24 human bladder cancer cells, both agents caused significant autophagy activation. Autophagy inhibition with CQ, 3-MA, or BAF A1 significantly reduced the viability of T24 and J82 cells when combined with gefitinib or lapatinib compared with each treatment alone, as well as improving the anti-clonogenic activity of the EGFR inhibitors in both cell lines. The cytoprotective role of autophagy was further confirmed by genetic inhibition of autophagy using ATG12-directed siRNA, which resulted in an increased antitumor effect of lapatinib and gefitinib.

In these studies with lapatinib, both cytoprotective and cytotoxic functions of autophagy were identified, making it difficult to reach unequivocal conclusions regarding whether autophagy inhibition might serve a useful clinical function.

### 2.2. ALK Inhibitors

#### 2.2.1. Brigatinib

Brigatinib is a small-molecule inhibitor for anaplastic lymphoma kinase (ALK) that was approved in 2017 for the treatment of ALK-positive NSCLC [56,57]. Unfortunately, limited information in the scientific literature is available on the role of autophagy in mediating the antitumor response to brigatinib. One study by Zhang et al. investigated the effect of brigatinib in a colorectal cancer cell model [58]. Brigatinib was capable of suppressing the proliferative potential of both ALK-positive H3122 and H2228 NSCLC cells, as well as ALK-negative A549 (NSCLC), Hep3B (HCC), Du145 (Prostate), and HCT116 (Colon) cell lines, suggesting that brigatinib possesses an ALK-independent antitumor potential. Interestingly, brigatinib induced ER stress, as evidenced by the elevated levels of ER stress markers PERK, p-PERK, IRE1α, p-IRE1α, and CHOP. Importantly, upon combining brigatinib together with the ER stress inhibitor, 4-phenylbutyrate (4-PBA), brigatinib-mediated apoptosis was abrogated, suggesting that the ER-stress response induced by brigatinib triggers apoptosis. Furthermore, this combination inhibited brigatinib-induced cytotoxicity and anti-proliferative effects. Brigatinib was also able to induce autophagy in the CRC cell lines, as shown by LC3I/II conversion, LC3II puncta formation, and the upregulated levels of ATG5, ATG7, and Beclin 1 proteins. The induction of autophagy was abrogated via ER stress inhibition using 4-PBA as well as IRE1α knockdown but not PERK knockdown, suggesting that brigatinib induces autophagy via the IRE1α/JNK signaling pathway in response to ER stress. Consequently, more specifically, brigatinib treatment increased the expression of FAM134B, a common ER-anchored receptor that is responsible for ER delivery into autophagosomes [59,60]. The latter observation was further confirmed by FAM134B knockdown, where the autophagy markers were suppressed upon combing FAM134B knockdown with brigatinib. These results confirmed that brigatinib induced ER-phagy, a form of selective autophagy that is mainly mediated by specific ER receptors, in which portions/fragments of the ER degraded within lysosomes/autophagic system [61,62,63], in CRC cell lines, but did not shed light on the functional form of this autophagy. 

Zhang et al. [58] investigated the role of ER-phagy in CRC cells using the autophagy inhibitors CQ, 3-MA, or BAF A1. The autophagy inhibitors together with brigatinib showed enhanced antitumor activity as compared with brigatinib alone, suggesting a cytoprotective role of autophagy in this system. The cytoprotective role of autophagy was further confirmed when autophagy was inhibited by a genetic strategy with ATG5, ATG7, or BECN1 knockdown, and autophagy inhibition increased the antitumor efficacy of brigatinib. Furthermore, autophagy/ER-phagy inhibition augmented brigatinib-induced apoptosis in CRC cells. These results were confirmed using nude mice subcutaneously inoculated with DLD-1 cells. Brigatinib treatment increased LC3II expression as detected by immunohistochemical staining; CQ in combination with brigatinib exhibited superior anti-neoplastic activity as compared with brigatinib alone, as demonstrated by increased apoptosis, reduced tumor size, and Ki67 staining. These results collectively support a cytoprotective role for brigatinib-induced autophagy in CRC cells, both in vivo and in vitro. 

#### 2.2.2. Lorlatinib

Lorlatinib is a third-generation/ATP-competitive small-molecule TKI that targets ALK [64]. Lorlatinib showed efficacy in overcoming resistance to first- and second-generation ALK tyrosine kinase inhibitors [65,66]. As is the case with brigatinib, limited data are available on any connection between autophagy and lorlatinib. Lu et al. showed that lorlatinib inhibited the proliferation of ALK-positive H3122 and H2228 NSCLC cells, as evidenced by their reduced clonogenic potential and precipitation of apoptosis [64]. Additionally, lorlatinib reduced total ALK protein expression levels and the phosphorylation of both AKT and mTOR, coupled with an autophagic response in both cell lines, as evidenced by the accumulation of autophagosomes, a reduction in p62/SQSTM1 levels, and increased LC3II levels [64]. The combination of lorlatinib with CQ or 3-MA significantly inhibited autophagic flux and increased the sensitivity of both cell lines to lorlatinib, indicative of the cytoprotective role of lorlatinib-induced autophagy. Finally, CQ was able to produce similar effects in vivo using a H3122 xenograft mouse model, where lorlatinib treatment in combination with autophagy inhibition reduced tumor growth, with significant tumor shrinkage and apoptosis significantly greater effects than for lorlatinib alone.

#### 2.2.3. Crizotinib

Crizotinib is a small-molecule inhibitor targeting c-Met, ALK, and ROS1 tyrosine kinases that is approved for NSCLC harboring EML4-ALK rearrangements [67]. You et al. [68] showed that crizotinib treatment induces autophagy in multiple lung tumor cell lines, including SPC-A1, A549, and H2228 cells, as shown by LC3 lipidation and p62/SQSTM1 degradation. Importantly, inhibiting autophagy using 3-MA or CQ sensitized SPC-A1 and A549 cells to crizotinib, while shRNA-mediated Beclin 1 depletion sensitized SPC-A1 cells to crizotinib via the direct promotion of apoptosis. You et al. [68] further validated their results in vivo using a SPC-A1 xenograft mouse model, where crizotinib-induced autophagy was effectively inhibited by HCQ and resulted in a significant reduction in tumor weight. Furthermore, autophagy inhibition induced apoptosis, as confirmed by elevated cleaved caspase-3 levels. These results confirm the cytoprotective role of autophagy induced by crizotinib in lung cancer. Mechanistically, inhibition of the STAT3 signaling pathway was involved in mediating the cytoprotective role of crizotinib-induced autophagy.

Ji et al. investigated whether autophagy induction may contribute to crizotinib resistance in lung cancer cells [69]. Autophagy levels were compared between both H3122-sensitive and H3122CR-1-resistant cells, where the resistant cells exhibited significantly higher levels of LC3II. Furthermore, crizotinib treatment caused a dose-dependent activation of autophagy in the resistant cells, confirmed by enhanced autophagosome formation. Importantly, inhibiting autophagy using CQ and BAF A1 increased the sensitivity of crizotinib-treated resistant cells. Additionally, autophagy inhibition resulted in significant apoptosis, suggesting that autophagy plays a cytoprotective role in this system and contributes to crizotinib resistance in lung cancer cells. The cytoprotective nature of crizotinib-induced autophagy was further confirmed in vivo using a mouse xenograft model of H3122CR-1-resistant cells. Again, the combination of CQ and crizotinib suppressed the tumor growth more effectively than crizotinib alone. Mechanistically, they showed that the levels of ALK are inversely correlated with crizotinib resistance. Specifically, when investigating the levels of phospho-ALK and total ALK in H3122 cells with varying sensitivity to crizotinib, the resistant cell line had the lowest levels with elevated autophagy. ALK knockdown using siRNA in the sensitive cell lines was followed by autophagy induction, suggesting a link between ALK and the autophagic machinery and that ALK downregulation is an initial step for autophagy activation. 

Mitou et al. investigated the potential contribution of autophagy to mediating sensitivity to crizotinib in lymphoma cells [70]. ALK inactivation via either crizotinib or siRNA-mediated ALK knockdown triggered autophagy in the ALK-positive large cell lymphoma cell lines, Karpas-299 and SU-DHL-1, as evidenced by acridine orange staining. Autophagy induction was further confirmed by electron microscopy, which showed autophagosome accumulation as well as the upregulation of several autophagy regulatory genes. Interestingly, exposing ALK-negative FEBD cells to crizotinib did not result in significant autophagy induction, in contrast to Ji et al. [69] findings, highlighting the need for further exploring the relation between ALK and autophagy. Importantly, combining CQ or 3-MA with crizotinib caused a synergetic reduction in cell viability compared with each drug alone. Similar results were obtained upon siRNA-mediated ATG7 knockdown, where autophagy interference caused a synergistic reduction in viability and clonogenicity of Karpas-299 and SU-DHL-1 cells when combined with crizotinib, suggesting a cytoprotective role of autophagy in this model. Mitou et al. [70] confirmed the cytoprotective role of the autophagic flux in vivo using a Karpas-299 xenograft mouse tumor model, where CQ in combination with crizotinib showed a significant reduction in tumor growth as compared with each drug alone. Furthermore, both necrosis and apoptosis were induced in tumors exposed to the combination. These results are consistent with observations by both Ji et al. [69] and You et al. [68] in lung cancer, confirming the cytoprotective role of crizotinib-induced autophagy.

In gastric cancer, Schroeder et al. showed that crizotinib induces apoptosis in MET-overexpressing SNU-5 and MKN45 cells but not in cells with wild-type or mutated MET [71]. Furthermore, mTORC1 gene expression was significantly downregulated upon crizotinib treatment in both cell lines, coupled with increased acridine orange staining and LC3I/II conversion, indicative of autophagy induction [71]. Combining CQ with crizotinib reduced crizotinib-mediated apoptosis in MET-overexpressed SNU-5 and MKN45 cells. Furthermore, genetic depletion of autophagy via ATD5/7 knockdown blocked apoptosis mediated by MET knockdown, indicating that autophagy plays a cytotoxic role in this system. 

The bulk of the experimental evidence is consistent with the premise that cytoprotective autophagy is the predominant form induced in response to crizotinib treatment, with the exception of the findings by Schroeder et al. in gastric cancer [71], suggesting that autophagy targeting may be a valid strategy for increasing the effectiveness of crizotinib therapy.

### 2.3. EGFR Inhibitors

#### 2.3.1. Gefitinib

Gefitinib is a first-generation, orally bioavailable, competitive, reversible EGFR TKI that interferes with tyrosine kinase signaling in tumor cells with mutated and hyperactive EGFR [72]. Gefitinib is approved for treating patients with metastatic NSCLC who have tumors with either EGFR exon 19 deletions or exon 21 (L858R) substitution mutations [73]. Gefitinib was shown to promote autophagy in lung cancer cell lines, as indicated by LC3II lysosomal localization, increased ATG5 and ATG7 expression at the mRNA or protein levels, and reduced phosphorylation of the PI3K/Akt/mTOR pathway [74,75]. Gefitinib was primarily associated with the induction of a cytoprotective form of autophagy. For example, Sugita et al. indicated that autophagy inhibition by clarithromycin through the inhibition of autophagy flux enhanced the cytotoxicity of gefitinib by interfering with gefitinib-induced autophagy [76]. Furthermore, due to concerns about off-target effects of clarithromycin, ATG5 silencing, which was accompanied by increased p62/SQSTM1 and a decrease in the LC3I/II conversion, resulted in a significant reduction in cell migration and invasion over and above that induced by gefitinib alone, as well as an increase in gefitinib sensitivity in NSCLC-resistant cell lines [77]. Moreover, Cheng et al. demonstrated a synergistic effect between gefitinib and MK-2206, a potent allosteric Akt inhibitor (an autophagy inhibitor) currently in Phase II trials [78], in glioma cell lines [79]. Akt inhibitors like MK-2206 may contribute to inducing a functional switch from autophagy to apoptosis. This transition could potentially elucidate the synergistic impact of MK-2206 on the cytotoxic activity of gefitinib. In the presence of MK-2206, concurrent elevation of both apoptosis and autophagy was observed in tumor cells treated with gefitinib for 48 h. Subsequently, 48 h after the co-treatments, a reduction in autophagic activity was observed, accompanied by a further activation of apoptosis. MK-2206 co-treatment showed increased Annexin V staining, indicative of apoptosis induction and upregulation of the pro-apoptotic protein Bim [79]. Furthermore, suppression of autophagy following transfection with a Beclin 1 siRNA significantly enhanced the cytotoxic activity of the combinatorial treatment [79]. These results suggest that autophagy inhibition can improve the cytotoxic effect of gefitinib in both glioma and NSCLC models. 

Autophagy is also implicated in the development of resistance against EGFR. Over time, patients may develop resistance to TKIs, leading to disease recurrence as a result of the emergence of secondary EGFR mutations such as T790M, the most frequent mutation for most patients receiving first-line therapy such as gefitinib [80,81]. It has been shown that the prolonged exposure of several lung cancer models to gefitinib is associated with upregulated levels of autophagy [74,82,83]. Han et al. compared gefitinib treatment in both sensitive and resistant lung cancer cell lines; interestingly, immunoblotting and immunofluorescent staining demonstrated an increase in both ATG5 and ATG7 levels and LC3II expression in resistant cell lines [74]. To confirm the cytoprotective role of autophagy, resistant cell lines were subjected to treatment with CQ or ATG5 or ATG7 knockdown, and both approaches for autophagy suppression augmented the growth inhibition induced by gefitinib [74]. In a recent study, Wu et al. discovered that the acquired T790M EGFR mutation leads to a significant reconfiguration of EGFR’s intracellular trafficking patterns in response to TKI treatment [84]. More specifically, autophagy is induced by several TKIs, including gefitinib, which results in EGFR degradation in TKI-resistant models. However, NSCLC cells carrying only primary EGFR mutations prompt the recycling of EGFR back to the cellular plasma membrane [84]. This resistance was highlighted by a four-fold decrease in the IC_50_ values for gefitinib when ATG12 gene silencing was performed in JIMT1 cells, which are HER2-resistant-positive breast cancer cells, indicative of the cytoprotective nature of gefitinib-induced autophagy [85].

Conversely, several studies have identified a potential cytotoxic effect of autophagy induction following EGFR-TKI treatment. For example, Zhao et al. suggested that gefitinib could promote both autophagy and apoptosis in lung cancer cells by blocking the PI3K/AKT/mTOR pathway, leading to tumor cell demise [86]. Furthermore, reduced autophagy has been linked to elevated ATG16-L1 β (autophagy-related 16-like 1) expression [87]. ATG16-L1 is a subunit of the autophagy-related ATG12-ATG5/ATG16 complex that retains exon 8 and is essential for LC3 lipidation and autophagosome formation [88]. The reduction of ATG16-L1 β reinstated autophagy activation and responsiveness to EGFR-TKI treatment by stimulating apoptosis [87]. When autophagy was hindered by administering the lysosomotropic agent BAF A1 to ATG16-L1 β-inhibited cells (transfected with siEx8), apoptosis was partially prevented, suggesting that in this experimental model system, autophagy contributes to the facilitation of cell death [87]. Consistent with this conclusion, the promotion of autophagy using the mTOR inhibitor rapamycin enhanced the responsiveness of resistant tumor cells to EGFR tyrosine kinase inhibition [89]. 

Yiqi Chutan Tang (YQCT) is a traditional Chinese medicine (TCM) formula developed for the treatment of NSCLC [90]. Studies have demonstrated its ability to suppress tumor growth in mice, mitigate drug resistance in lung cancer cells, extend the median survival time, and alleviate chemotherapy-related fatigue in patients with NSCLC [91,92]. In the context of treating gefitinib-resistant NSCLC cell lines with a combination of gefitinib and YQCT, a significant increase in the protein levels of ATG3 and ATG12 in H1975 NSCLC cells after 48 h was observed, as compared with cells treated with gefitinib alone [93]. This finding suggests that YQCT enhances the autophagic process triggered by gefitinib, underscoring the importance of autophagy as a potential molecular mechanism through which YQCT can mitigate gefitinib-induced drug resistance [93].

The role of gefitinib-induced autophagy is not limited to tumor models. A study by Luo et al. on gefitinib-induced hepatotoxicity showed that gefitinib activates excessive autophagic degradation of COX6A1 (a protein involved in maintaining liver homeostasis [94]) in hepatocytes, ultimately resulting in aberrant apoptosis, a primary feature of liver injury [95]. Notably, this autophagy-mediated apoptosis is dependent on polo-like kinase 1 (PLK1) [96]. Subsequently, exposure to gefitinib results in an increase in PLK1 expression levels in human liver cell lines and human primary hepatocytes. Furthermore, inhibition of PLK1 activity with BI-2536 (a PLK1 inhibitor) or depletion of PLK1 by RNAi in mice could restore the LC3II and COX6A1 levels altered by gefitinib [97]. These results suggested that autophagy inhibition might contribute to the mitigation of gefitinib-induced hepatotoxicity.

#### 2.3.2. Erlotinib

Erlotinib is a first-generation EGFR TKI approved for the treatment of NSCLC. It is being utilized as a first-line treatment in patients with a sensitizing mutation in the tyrosine kinase domain, such as exon 19 deletion or L858R [98]. The function(s) of erlotinib-induced autophagy are currently uncertain. Li et al. reported the cytoprotective role of autophagy in NSCLC [99]. In EGFR-resistant lung cancer cells (A549 and NCI-H1299 cells), erlotinib promoted the conversion of LC3I to LC3II in a dose- and time-dependent manner. Furthermore, phosphorylation of AKT, mTOR, and p70S6K in both A549 and H1299 cells was significantly reduced, leading to decreased activity of these proteins [74]. The activity of the AKT, mTOR, and p70S6K pathways inhibits the autophagic process and is frequently related to prosurvival effects, and the suppression of this pathway’s activity promotes autophagy [100]. Erlotinib increased ATG5 and ATG7 expression, consistent with the promotion of autophagy. Growth inhibition induced by erlotinib in A549 cells was enhanced following autophagy inhibition by knockdown of ATG5 or ATG7. In these studies, erlotinib-induced autophagy clearly demonstrated a cytoprotective function [74]. 

In contrast to the findings by Li et al. [99], Jiang et al. showed that autophagy enhanced the activity of erlotinib. In A549 cells, combined treatment of sertraline, an antidepressant, and erlotinib significantly increased LC3II accumulation and decreased p62/SQSTM1 levels. Increased autophagic flux was confirmed by TEM. Inhibition of autophagy through ATG5 knockdown, shRNA-mediated downregulation of Beclin 1, or using CQ, 3-MA, and BAF A1 all led to decreased cytotoxicity of the combined treatment [101], whereas sertraline enhanced erlotinib-induced autophagy and improved the therapeutic efficacy of erlotinib in NSCLC cells. Here, autophagy clearly demonstrated a cytotoxic function.

The differences in the outcomes of the studies by Li et al. [99] and Jiang et al. [101] might suggest the autophagic switch that we have reported previously, where one form of autophagy is converted to a different form by genetic or pharmacologic manipulations [102]. In this case, erlotinib alone induced cytoprotective autophagy, while in combination with sertraline, a cytotoxic form emerged. These results further confirm that autophagy is dependent on the chemical nature of the compound(s) under investigation, in addition to the cell line/tumor model.

#### 2.3.3. Osimertinib

Osimertinib, a third-generation irreversible EGFR-TKI, has shown efficacy in advanced NSCLC patients harboring T790M, which is the most frequently acquired resistance mechanism after treatment with first-generation EGFR-TKIs [103,104,105]. Moreover, osimertinib showed superior efficacy to that of first- or second-generation EGFR-TKIs in the first-line treatment of EGFR-mutant advanced NSCLC [106]. Osimertinib has been shown to induce autophagy in ovarian, hepatocellular, colon, and NSCLC cell lines [107]. Furthermore, autophagy has been observed in osimertinib-treated NSCLC cells that harbor wild-type EGFR and in cells that harbor EGFR mutations, L858R/T790M, through increased levels of LC3 and a reduced expression of p62/SQSMT1 [108]. 

In a study by Chen et al., osimertinib triggered autophagy, characterized by substantial increases in Beclin 1, LC3II/I ratio, and a decrease in p62/SQSTM1 [109]. Upon the addition of spautin-1 or 3-MA, both early autophagy inhibitors [110,111], to osimertinib-treated cells, a substantive reduction in the ratio of LC3II/I was evident [109]. This reduction was accompanied by significantly decreased levels of Beclin 1, ATG7, and ATG5, along with an increased level of p62/SQSMT1 in PC-9GR (T790M mutated lung cancer) cells. Importantly, these changes resulted in a notable augmentation of osimertinib’s inhibitory effect on the growth of PC-9GR cells, suggesting a cytoprotective role of autophagy in response to osimertinib [109].

Work conducted by Li et al. demonstrated that the combined utilization of osimertinib alongside CQ significantly reduced the expressions of ALDH1A1, SOX2 proteins, and the CD133/CD44 positive cell population in osimertinib-resistant cells, highlighting a critical role of autophagy in mediating the stemness of ovarian cancer cells [41]. Moreover, inhibition of autophagy through ATG5 knockdown resulted in a decrease in the CD44 + CD117+ cell proportion, concurrently diminishing both the chemoresistance and tumorigenic potential of ovarian cancer stem cells [41]. These observations underscore the contribution of autophagy to maintaining the stemness of cancer cells, leading to heightened therapeutic resistance.

In related work, targeted inhibition of autophagy via siRNA-mediated knockdown of Beclin 1 led to the complete suppression of stem cell-like characteristics, including diminished levels of key stemness markers such as SOX2, ALDH1A1, and CD133/CD44 in HNSCC cells [112]. Li et al. [41] demonstrated the key regulatory role of autophagy in driving osimertinib resistance by modulating stem cell-like properties, with Beclin 1 playing a pivotal role in this process. Moreover, in vivo experiments involving tumor xenografts revealed that the combined treatment of osimertinib and CQ notably suppressed tumor growth compared to treatment with osimertinib alone [41]. These results, again, confirm a cytoprotective role for osimertinib-induced autophagy.

Osimertinib has been documented to induce cytoprotective autophagy in colon cancer [113]. Apoptotic cell death induced by osimertinib was intensified in the presence of pharmacological inhibitors of autophagy, marked by increased levels of cleaved PARP and cleaved caspase 3 [113]. Despite the preponderance of evidence in support of the premise that osimertinib promotes primarily the cytoprotective form of autophagy, in certain cases, a cytotoxic form of autophagy was identified. For example, a study by Sazuki et al. revealed that treating NSCLC cells with lurasidone, an antipsychotic, sensitized these cells to osimertinib by reducing the expression of survivin [114,115], which is known for its anti-apoptotic function and as a negative regulator for autophagy. Immunoblotting analysis following lurasidone-only treatment revealed an increase in LC3II expression, suggesting induction of autophagy. To confirm the role of autophagy, NSCLC cells were treated with an autophagy inhibitor, 3-MA, followed by osimertinib [114]. The experimental results indicated reduced sensitivity of the lurasidone-treated cells to osimertinib when autophagy was inhibited, underscoring the potential link between lurasidone-induced sensitivity of NSCLC cells to osimertinib and enhanced autophagy [114]. Cytotoxic autophagy, which refers to a form of autophagic process in which the cell actively engages in the degradation of its own components, leads to programmed cell death. This type of autophagy plays a direct role in inducing cell death and is often triggered by cellular stress or damage. Whereas autophagy facilitating cell death refers to a role of autophagy where it contributes to cell death without being the primary driver. Instead of actively inducing cell death, autophagy in this context plays a supporting role, responding to cellular stressors and enhancing the overall process of cellular demise [116,117].

#### 2.3.4. Dacomitinib

Dacomitinib, categorized as a second-generation EGFR TKI, irreversibly inhibits the human EGFRs (HERs), encompassing HER-1/EGFR, HER-2, and HER-4 [118]. Numerous studies have indicated that dacomitinib demonstrates enhanced anti-cancer efficacy compared with erlotinib in models of NSCLC, whether the tumors are responsive or resistant to erlotinib [119,120]. Previous research (discussed above) has proposed that first-generation EGFR TKIs, such as erlotinib and gefitinib, are likely to elicit a cytoprotective autophagic response [99]. Moreover, autophagy was shown to contribute to resistance against the inhibitory effects on proliferation and cell cycle progression induced by these TKIs. Similarly, the role of dacomitinib-induced autophagy has been explored in multiple studies. For example, the autophagic impact of dacomitinib on NCI-H1975 cells and ovarian cancer cell lines revealed an elevated expression of LC3II in response to dacomitinib treatment [121,122]. 

Tang et al. found that the downregulation of ATG7, ATG5, or Beclin 1 reversed the dacomitinib-induced upregulation of LC3II expression, indicating that dacomitinib has the ability to induce autophagic flux in NCI-H1975 cells [121]. The dacomitinib-induced inhibitory effect on proliferation was enhanced when autophagy was inhibited through pretreatment with BAF A1 or the silencing of ATG7 [121], indicating that the autophagy induced by dacomitinib counteracts its inhibitory effect on proliferation in NCI-H1975 cells. Upon autophagic inhibition, dacomitinib produced a further elevation of cleaved PARP and cleaved caspase-3 levels (i.e., increased apoptosis) [121]. These findings indicate that dacomitinib induces a cytoprotective autophagy response in NCI-H1975 cells. In non-tumor cell models, dacomitinib also triggers a protective form of autophagy, as shown in two experimental models of pulmonary arterial hypertension (PAH) as well as in primary rat pulmonary smooth muscle cells (PASMCs) [123]. 

#### 2.3.5. Mobocertinib 

In 2021, FDA approval was granted to mobocertinib for adult patients with locally advanced or metastatic NSCLC featuring EGFR exon 20 insertion mutations, identified through an FDA-sanctioned test, whose condition has advanced following platinum-based chemotherapy [124]. Daily oral administration of mobocertinib at well-tolerated doses led to tumor regression in a Ba/F3 ASV xenograft mouse model [125]. Mobocertinib’s mechanism of action is similar to that of second- and third-generation EGFR-TKIs, exerting its effects through irreversible binding to the cysteine residue at position 797 in EGFR [125]. In addition to targeting EGFRex20ins, mobocertinib has also shown efficacy against common *EGFR* mutations, including L858R and exon 19 deletions, in preclinical studies [125]. Nevertheless, to our knowledge, the involvement of autophagy in mobocertinib-induced cell death in NSCLC has not been explored.

### 2.4. VEGFR Inhibitors

Vascular endothelial growth factor receptor 2 (VEGFR-2) serves as the principal mediator in the signal transduction of VEGF/VEGFR, playing a pivotal role in the stimulation of tumor angiogenesis [126]. The phosphorylation of VEGFR-2 initiates the activation of the Raf-1/Mitogen-Activated Protein Kinase/Extracellular Signal-Regulated Kinase (MAPK/ERK) signaling cascade. This molecular pathway ultimately induces angiogenesis, heightened vascular permeability, as well as tumor proliferation and migration [127]. Consequently, the inhibition of the VEGFR-2/VEGF signaling axis is recognized as a pivotal strategy in cancer treatment [128]. Currently, several VEGFR-2 inhibitors, approved by the FDA, are employed as chemotherapeutic agents [129]. Nevertheless, the emergence of drug resistance poses a challenge, diminishing drug efficacy and escalating toxicity, leading to unwarranted side effects. Autophagy has been implicated as one mechanism that contributes to resistance against various monoclonal antibodies that target the VEGFR signaling pathways [34]. 

#### 2.4.1. Vandetanib

Vandetanib is a second-generation small-molecule kinase inhibitor with multi-target activity, primarily exerting inhibition of VEGFR 1, 2, and 3, as well as EGFR, and rearranging during transfection (RET) [130]. In 2011, the FDA granted approval for vandetanib in the management of medullary thyroid carcinoma (MTC) [131], a drug that has demonstrated encouraging outcomes in clinical trials for advanced NSCLC [132,133]. Zhou et al. studied vandetanib in the Calu-6 NSCLC cell line. Through Western blot analysis, a notable rise in the expression of the autophagic marker LC3-II was observed in response to vandetanib treatment. This increase exhibited a concentration-dependent pattern, suggesting that vandetanib induces autophagy in Calu-6 cells [134]. Furthermore, co-treatment of vandetanib and an autophagy inhibitor (3-MA or CQ) significantly reduced the viability of Calu-6 cells [134]. Vandetanib treatment resulted in the activation of the PI3K-AKT-mTOR pathway rather than its suppression, indicating that vandetanib-induced autophagy occurs independently of PI3K-AKT-mTOR [134]. Notably, an increase in reactive oxygen species (ROS) levels was observed in Calu-6 cells following vandetanib treatment. ROS have been found to play an integral role in autophagy induction [135]. A ROS scavenger was introduced to vandetanib-treated cells, and inhibition of the formation of GFP-LC3 puncta was observed. The inhibition of ROS enhanced sensitivity to vandetanib in Calu-6 cells through the inhibition of autophagy [134]. These findings are corroborated by the studies of Shen et al., who investigated the role of vandetanib-induced autophagy in glioblastoma cell lines (U251, U87MG) [136]. Following knockdown of ATG7 and Beclin 1 expression using siRNA, autophagy was suppressed, as indicated by interference with the vandetanib-induced increase in LC3 lipidation. Moreover, the total number of surviving cells substantially decreased following knockdown of ATG7 and Beclin 1 along with vandetanib treatment [136]. However, contrary to the previous study, Shen et al. suggested that inhibition of the PI3K/Akt/mTOR pathway is required for the induction of autophagy after vandetanib treatment [136]. The combination of HCQ and vandetanib was pursued further in experiments where vandetanib was encapsulated in liposomes to assess the synergistic anti-glioma effects both in vitro and in vivo. HCQ significantly inhibited autophagy and dramatically enhanced the anti-glioma abilities of vandetanib [137]. The above studies highlight the potential of autophagy inhibition in conjunction with vandetanib for improved cancer treatment. 

#### 2.4.2. Cabozantinib

Cabozantinib is a small-molecule second-generation tyrosine kinase inhibitor that also targets c-Met (mesenchymal-epithelial transition) and VEGF [138,139] and has received approval for the treatment of advanced renal RCC and differentiated thyroid cancer (DTC). Renal cell carcinoma (RCC) cells are characterized by the upregulation and increased activity of c-Met [140]. Binding of c-Met to its ligand, hepatocyte growth factor (HGF), triggers pathways that promote tumor growth [141]. Additionally, this activation of c-Met has the potential to induce therapeutic resistance in RCC [142]. Scott et al. [143] observed elevated protein levels of ATG3, LC3, and Beclin 1 as well as a decrease in SQSTM1/p62 as early as seven days following the treatment of colorectal cancer cell lines with cabozantinib [143]. Following the increase in autophagy, HCT116 cells were cotreated with cabozantinib and autophagy inhibitors such as CQ and SBI-0206965, a ULK1 inhibitor [144], resulting in a significant increase in apoptosis and suggesting that cabozantinib-induced autophagy exhibits a cytoprotective function [143]. However, upon combining cabozantinib with another compound led to a different autophagic response in studies by Rawat et al. [145], where cabozantinib in combination with the natural product, honokiol [145], elevated reactive oxygen, triggered apoptosis, and induced a cytotoxic form of autophagy in renal cancer cell lines.

#### 2.4.3. Sunitinib and Sorafenib

Sunitinib and sorafenib, second-generation TKIs, have been formulated as orally administered small molecules targeting receptors such as VEGFR and PDGFR [146]. Sunitinib and sorafenib’s ability to selectively inhibit these receptors contributes to their therapeutic efficacy in impeding aberrant signaling pathways associated with cancer growth and angiogenesis [147,148]. However, the rapid emergence of both intrinsic and acquired resistance poses a significant challenge, limiting their clinical efficacy [149,150]. 

Ikeda et al. observed a notable rise in LC3-II levels and a decrease in p62/SQSTM1 after exposing pheochromocytoma cells (PC12 cells) to sunitinib. Following treatment, immunofluorescent imaging demonstrated a rise in the punctate distribution of LC3-II [151]. Furthermore, sunitinib-induced autophagy was mitigated by ATG13 knockdown. Conversely, inhibiting autophagy using ATG13-targeting siRNA or ammonium chloride enhanced both sunitinib-induced apoptosis and anti-proliferative effects [151]. However, when sunitinib was combined with ATG5 or ATG7 knockdown in BON1 pancreatic neuroendocrine cell lines, negligible activation of apoptosis was observed. In contrast, combining sunitinib with autophagy inhibitors targeting lysosomes, such as CQ, Baf A1, or LAMP2 knockdown, resulted in apoptosis induction, suggesting that these autophagy inhibitors might exert autophagy-independent effects [152]. 

Further evidence for the cytoprotective function of sunitinib is provided by the observation that the combination of CQ and sunitinib increased cytotoxicity across various cancer cell lines (MCF-7, T-47D, Hela, Caco-2, HCT116, HepG2, HEp-2, PC3), as evidenced by combination and concentration reduction indices [153]. Sunitinib induced autophagy in Ehrlich ascites carcinoma cells (EAC) implanted in Swiss albino mouse models through upregulation of Beclin 1, a process blocked by CQ, as indicated by elevated p62/SQSTM1 levels [153]. Moreover, CQ enhanced sunitinib-induced apoptosis, decreasing survivin levels and increasing caspase-3 activity [153]. In summary, these findings suggest that CQ enhances sunitinib cytotoxicity synergistically by inducing apoptosis and suppressing autophagic and angiogenic processes. 

In a study involving bladder cancer models, sunitinib was also found to activate the autophagic process, as indicated by the conversion of LC3I to LC3II, increasing the LC3II/LC3I ratio [154]. However, despite this activation, treatment with sunitinib led to the accumulation of p62/SQSTM1 protein in bladder cancer cells, implying impaired lysosomal degradation or incomplete autophagolysosome formation [154]. Consequently, autophagy disruption resulted in the accumulation of p62/SQSTM1, indicating incomplete autophagy [154]. Incomplete autophagy, also known as lysosomal sequestration, has been described as a mechanism conferring sunitinib resistance [155].

With regard to sorafenib, Shi et al. observed that sorafenib induces both apoptosis and autophagy in human HCC cells by upregulating IRE1 signals associated with endoplasmic reticulum (ER) stress [156]. Additionally, sorafenib can trigger autophagy by activating the Akt pathway or inhibiting mTORC1 [157,158]. Furthermore, in the study by Tai et al., sorafenib activated autophagy by disrupting the interaction between Beclin 1 and myeloid cell leukemia-1 (Mcl-1) [159]. Sorafenib has been found to promote LC3 lipidation, a clear indication of autophagy induction in hepatocellular cancer [159,160]. In addition, sorafenib modulates the expression of multiple autophagy markers, such as Beclin1, ATG5, and ATG12, in HCC cells [160]. Beclin 1 expression is increased in a time-dependent fashion in Hep3B cells [161]. Sorafenib can moderately induce Beclin 1 and ATG-5 expression, whereas p62/SQSTM1 expression is markedly decreased in PLC-5 cells [159].

In RCC (786-0 and A489 lines), sorafenib activated autophagy, as evidenced by p62/SQSTM1 degradation, Beclin 1/autophagy protein 5 (ATG-5) upregulation, and LC3I/II conversion [162]. The exogenous introduction of miR-30a (potent inhibitors of autophagy [163]) into RCC cell lines inhibited Beclin 1 expression and enhanced sorafenib-induced cytotoxicity [162]. Ling et al. showed that the combined treatment of metformin and sorafenib significantly inhibits the mTOR pathway [164], promoting autophagy. This combination led to suppressed proliferation and enhanced tumor inhibition in HCC. Following treatment of the HCC cells with CQ, an increase in LC3II and enhanced sensitivity of HCC cells to metformin and sorefenib-induced apoptotic cell death were observed [164].

Nevertheless, sorafenib-induced autophagy does not always serve a cytoprotective role, as other studies in HCC [165] have suggested. For example, Tai et al. showed autophagy induction in HCC-bearing mice after treatment with a kinase-independent derivative of sorafenib, SC-59 [159]. A significant induction of SHP-1 activity was observed in SC-59-treated samples [159]. SHP-1 has been proposed as a candidate tumor suppressor gene in lymphoma, leukemia, and other cancers, as it functions as an antagonist to the growth-promoting and oncogenic potentials of tyrosine kinase [166]. Both sorafenib and SC-59 effectively suppressed tumor growth in PLC5 tumors. This finding was accompanied by the downregulation of P-STAT3, increased SHP-1 activity, and the induction of autophagy, indicating that sorafenib and SC-59 activate autophagy in HCC. Furthermore, with co-treatment with bafilomycin A1 and CQ, there was a significant reduction in the effect of sorafenib on cell viability. Moreover, the higher levels of autophagy induced by SC-59 were correlated to the anti-HCC effect in vitro and in vivo, which suggests a cytotoxic function of autophagy and indicates that autophagy works upstream of the growth suppressive effects of the drug. 

Sunitinib and sorafenib treatment have both been found to induce autophagy in thyroid cancer cell lines [167]. Upon ATG5 silencing, the antiproliferative effects of both sunitinib and sorafenib decreased [167]. Furthermore, when thyroid cancer cells were then treated with everolimus (an autophagy inducer similar to rapamycin), this resulted in an increase in the antiproliferative effect of both sunitinib and sorafenib [167]. These results suggest that sunitinib and sorafenib induce the cytotoxic form of autophagy.

Finally, the sole study investigating the potential association between sorafenib and autophagy in patients focused on refractory or relapsed lymphoproliferative disease [168]. Findings from this study revealed that non-responsive patients had lower baseline levels of LC3-II compared with responders. Moreover, patients demonstrating a positive response to sorafenib exhibited a more significant reduction in LC3 expression after one month of treatment [168]. These findings suggest a potential role for autophagy inhibition with sorafenib treatment.

#### 2.4.4. Regorafenib

Regorafenib is a multi-kinase inhibitor that targets PDGFR-β, VEGFR1-3, FGFR, and oncogenic receptor tyrosine kinases (RET, KIT, and RAF) and is indicated for the treatment of colorectal cancer and gastrointestinal tumors [169,170]. Regorafenib, a structural analogue of sorafenib, has also been approved for the treatment of patients with advanced HCC previously treated with sorafenib [171]. Autophagy induction has been observed in hepatoma cells (Hep3B) treated with regorafenib, marked by an increase in both Beclin 1 and LC3-II and a decrease in Bcl-2 levels [161]. These findings are supported by a study in HepG2 and Hep3B cell lines, where regorafenib induced pro-death autophagy by inhibiting AKT and mTOR signaling. Moreover, the observed cell death triggered by regorafenib was notably mitigated with the administration of 3-MA or CQ, implying that the regorafenib-induced HepG2 cell death was partially dependent on autophagy [172]. 

Jiang et al. observed an increase in LC3II accumulation in glioma cells and mouse xenografts in response to regorafenib treatment [173]. Treatment of glioma cells with regorafenib in combination with CQ significantly aggravated regorafenib-induced growth inhibition, whereas the combination with 3-MA markedly restored cell growth [173]. These findings suggest that regorafenib inhibits GBM cell growth by promoting autophagosome accumulation. Knockdown of ATG5 or ATG7 reduced the sensitivity of glioma cells to regorafenib treatment. These findings suggest that regorafenib treatment inhibits GBM cell growth by promoting autophagosome accumulation when used in combination with CQ; however, autophagy inhibition reduced regorafenib sensitivity, suggesting a cytotoxic role [173]. 

Regorafenib treatment was also tested in combination with anti-annexin A3 monoclonal antibodies (anti-ANXA3 mAb) [174]. Annexin A3 (ANXA3) plays a critical role in promoting aggressive cancer and stem cell-like properties in HCC and is involved in mediating the activation of autophagy and attenuation of PKCd (PRKCD)/p38-dependent apoptotic signaling [175]. Co-treatment of regorafenib and anti-ANXA3 mAb in sorafenib-resistant HepG2 xenografts led to decreased levels of LC3-II expression and autophagosome formation and an increase in apoptotic cells [174]. The above findings suggest a cytoprotective role for regorafenib-induced autophagy, in contrast to the studies by Jiang et al., Han et al., and Carr et al. However, genetic knockdown is needed for further clarification of the nature of regorafenib-induced autophagy [176]. 

#### 2.4.5. Pazopanib

Pazopanib, a second-generation TKI approved for the treatment of RCC and soft tissue sarcoma (STS), inhibits VEGFR, PDGFR, fibroblast growth factor receptor (FGFR), and stem cell receptor (c-Kit) [177]. Pazopanib has been found to induce the accumulation of LC3II in bladder cancer (BC) cells and to increase the LC3II/LC3I ratio, accompanied by a progressive degradation of p62/SQSTM1. Both the LC3II and p62/SQSTM1 levels increased after treatment with bafilomycin A1, implying a disruption in the autophagic flux. Furthermore, pazopanib induced cell death in BC cells, which was reversed by 3-MA [154], suggesting a cytotoxic role for pazopanib-induced autophagy.

#### 2.4.6. Lenvatinib

Lenvatinib is a multitargeted inhibitor that suppresses VEGFR 1–3, FGFR 1–4, and PDGFR α, as well as the proto-oncogenes RET and KIT [178]. Lenvatinib regulates the phosphorylation of ERK1/2 and mTOR in a dose-dependent manner, inducing autophagy in K1 and BCPAP cells [179]. Blocking autophagy with CQ and genetically knocking down autophagy by ATG-7 with siRNA effectively suppressed the proliferation of PTC (papillary thyroid cancer) cells and enhanced lenvatinib’s therapeutic effectiveness [179]. Co-treatment with lenvatinib and CQ resulted in a more pronounced reduction in VEGFA levels in the K1 and BCPAP cell supernatants, and xenograft tumor models confirmed these findings [179], indicative of the cytoprotective nature of lenvatinib-induced autophagy. However, Ye et al. found that lenvatinib treatment suppressed autophagy in gallbladder cancer cell lines (GBC-SD and NOZ). Western blot analysis revealed a decreased LC3II/I ratio and increased expression of p62/SQSTM1 following treatment with lenvatinib [180].

#### 2.4.7. Tivozanib

Tivozanib is a potent and selective TKI approved in 2021 for relapsed or refractory advanced renal cell carcinoma following two or more prior systemic therapies. It blocks VEGFR-1, -2, and -3 and inhibits angiogenesis and vascular permeability in tumor tissues [181]. The inhibitory effect on VEGFRs is stronger compared with other previously used TKIs in metastatic renal cell carcinoma (mRCC) [182]. However, there is essentially no information relating to autophagy in response to tivozanib treatment. 

#### 2.4.8. Axitinib

Axitinib, an oral second-generation TKI, is distinguished by its notable specificity for VEGF receptors. Axitinib effectively inhibits VEGF receptor subtypes 1, 2, and 3. In contrast to several other TKIs, axitinib exhibits limited activity towards alternative receptors, such as KIT and platelet-derived growth factor receptor (PDGFR) [183]. In a study conducted by Lin et al. (2020), it was documented that axitinib demonstrates effectiveness as a second-line therapeutic agent for individuals with advanced HCC who have experienced treatment failure with sorafenib [184]. In a comprehensive study, the analysis of more than 200 autophagy-related genes revealed that axitinib did not induce any significant modulation in the expression of these genes, as demonstrated by gene profiling [185]. Moreover, a constructed kinase–kinase inhibitor regulatory network in a specific study failed to report any instances of axitinib exerting regulatory effects on autophagy [186]. The varying impact of tyrosine kinase inhibitors on autophagy, excluding axitinib, may stem from distinct molecular structures and target profiles. Further studies are warranted to elucidate the precise role of autophagy in axitinib treatment.

### 2.5. BCR–ABL 

The Philadelphia chromosome (Ph+) results from a reciprocal translocation between chromosomes 9 and 22 that juxtaposes the BCR and ABL genes. The BCR-ABL fusion protein drives the malignant proliferation of myeloid stem cells and has been shown to be a major pathogenetic mechanism in chronic myelogenous leukemia (CML) [187]. In 2001, the FDA approved the ABL inhibitor imatinib for treating CML, which became the first small-molecule targeted oncology drug. However, imatinib has poor efficacy in advanced CML and is highly susceptible to resistance due to mutations in ABL, especially mutations T315I and L248V [188,189]. To address resistance, second-generation BCR-ABL inhibitors, nilotinib, dasatinib, and bosutinib, were developed. However, these drugs have not overcome the resistance conferred by the T3151 mutation. Ponatinib was unexpectedly identified as an effective BCR-ABL inhibitor against the ABL T3151 mutation and was approved by the FDA in 2012. Still, the poor kinase selectivity of ponatinib has limited its long-term clinical use [190]. Researchers have found that these drugs also have clear therapeutic effects on, for example, gastrointestinal stromal tumors (GISTs) and melanomas arising from mutations in receptor tyrosine kinases, but the inevitable resistance problems associated with these drugs have limited their clinical use.

#### 2.5.1. Imatinib

Numerous studies have identified autophagy in imatinib-treated CML and found that pharmacological inhibitors or RNA interference of essential autophagy genes enhanced cell death and promoted apoptosis induced by imatinib in cell lines and primary CML cells [191,192,193,194,195,196,197]. The GCA (grancalcin)-TRAF6-ULK1 autophagy regulatory axis is associated with imatinib resistance [198]. Cao et al. also reported that silencing of heme oxygenase-1 (HO-1) expression in K562-resistant cells resulted in suppression of autophagy and increased susceptibility to imatinib [199]. Results from the first clinical trial of an autophagy inhibitor with imatinib also showed that in patients with chronic phase chronic myeloid leukemia (CP-CML) who had been treated with imatinib for more than 12 months and had tolerated imatinib for more than 12 months, ‘successes’ (successes: a ≥0.5 log decrease in BCR-ABL1 qPCR levels after 12 months of imatinib treatment) at 24 months were higher in the HCQ combined with imatinib group than in the imatinib group (NCT01227135) [200]. These studies all suggest that combining autophagy inhibitors with imatinib may prove to have clinical value.

Contrary to these findings, some studies have come to the opposite conclusion: imatinib-induced downregulation of the Bcr-Abl protein is dependent on autophagy, and therefore inhibition of autophagy would instead attenuate the therapeutic effect of imatinib. Elzinga et al. found that autophagy was required for the decline of BCR-ABL protein and that siRNA-mediated knockdown of autophagy regulators (Beclin 1/ATG7) as well as the pharmacological inhibition (3-MA) of autophagy reduced BCR-ABL/LC3 co-localization in both K562 and CML patient cells [201]. Sheng et al. also demonstrated that imatinib-induced autophagy is a consequence of inhibition of the BCR-ABL/PI3K/AKT/FOXO4/ATF5/mTOR pathway [202]. That is, this ability to downregulate Bcr-Abl protein levels by inducing autophagy may be another important feature of imatinib activity. It may therefore occur that the combination of autophagy inducers with imatinib promotes cell death, a situation that, as described above, is independent of the function of autophagy and is associated with enhanced inhibition of BCR-ABL by imatinib. For example, Li et al. found that 2-Deoxyglucose (2-DG), a glucose analog that is an inhibitor of glucose metabolism, promotes CML cell death by inducing autophagy. Co-treatment with 2-DG and imatinib induced synergistic inhibitory effects in the KBM5 cell line and the BCR-ABL T315I mutant (KBM5-T315I) cell lines [203]. In short, these studies suggest that caution should be exercised when attempting to combat BCR-ABL-dependent imatinib resistance by inhibiting autophagy in CML treatment [204]. 

In addition to CML, imatinib has a therapeutic effect on gastrointestinal stromal tumors (GISTs), and autophagy is often detected during treatment [205,206]. Differing from CML, the role of imatinib-induced autophagy in GISTs has been reported more consistently. Inhibition of autophagy using RNAi-mediated silencing of ATG7 and ATG12 or CQ promotes GIST cell death in vitro and in vivo [207]. Recently, Ni et al. found that both CQ and bafilomycin A1 had the potential to increase the sensitivity of GFRA1-positive GIST-882 cells activated by rGDNF to imatinib. Thus, they suggested that GIST cells acquired resistance to imatinib by enhancing cellular autophagy, which is regulated by the GDNF-GFRA1 axis [208]. Subsequently, Gao et al. further demonstrated that a USP13 inhibitor causes ATG5 degradation and that the combination of the USP13 inhibitor with 3-MA enhances the efficacy of imatinib in a mouse xenograft model derived from GIST cells, which also indicates that imatinib resistance is associated with a cytoprotective role of autophagy [209]. Similarly, Zheng et al. found that CQ in combination with imatinib promoted apoptosis in imatinib-resistant GIST cells [210]. Chen et al. made further findings that the resistance of GIST cells to imatinib may be related to the interaction between miR-30a, which inhibits autophagy, and Beclin 1 [163]. 

To explore the therapeutic potential of imatinib, preclinical studies conducted on other tumors found that the role played by imatinib-induced autophagy was not entirely consistent. Different stages of autophagy inhibition may apparently exhibit different effects on imatinib activity in malignant gliomas. Suppression of imatinib-induced autophagy by 3-MA or with small interfering RNA against ATG5, inhibiting autophagy at an early stage, lessened the imatinib-induced cytotoxicity. In contrast, inhibition of autophagy at a late stage by vacuolar-type H^+^-ATPase inhibitors bafilomycin A1 or RTA 203 enhanced imatinib-induced cytotoxicity in U87-MG and U373-MG cells through the induction of apoptosis following mitochondrial disruption [211]. In addition to these observations, studies have shown that imatinib is an autophagy inhibitor for HCC cells. Xiao et al. found that imatinib treatment increased the levels of both p62/SQSTM1 and LC3 in HCC cells and HCC xenografts. Scanning confocal microscopy analysis with a mRFP-GFP-LC3 reporter, in addition to transmission electron microscopy analysis, revealed that imatinib suppressed the autophagic flux by blocking the formation of autolysosomes [212]. Similar results were reported by Roos and their colleagues. Imatinib raised the lysosomal pH and impaired lysosomal proteolytic function but increased the p62/SQSTM1 protein level [213]. 

These studies suggest that although autophagy plays a protective role in imatinib-induced cell death in CML, the combination of autophagy inhibitors (especially CQ) with imatinib needs to be evaluated more cautiously. As imatinib-induced autophagy is also necessary for BCR-ABL to be suppressed, inhibition of autophagy may weaken the inhibitory effect of imatinib on the BCR-ABL protein and subsequently reduce its efficacy. In addition, autophagy is not always induced by imatinib treatment, and there is potential to inhibit autophagy when imatinib treats other types of tumors in addition to CML.

#### 2.5.2. Bosutinib

Bosutinib is a second-generation tyrosine kinase inhibitor and a dual inhibitor of SRC and ABL kinases. Bosutinib was approved by the FDA for the treatment of CML in 2012 because of its ability to inhibit the BCR/ABL fusion gene product and its anticancer effects, which have been observed in imatinib-resistant CML [214]. Bosutinib has also been shown to inhibit the growth of solid tumors such as pancreatic, breast, prostate, and melanoma in preclinical models [215]. Studies on bosutinib-induced autophagy are limited, with only Noguchi et al. finding that bosutinib increased LC3II levels in melanoma cells. However, studies combining bafilomycin A1 and bosutinib showed that bafilomycin A1 did not further enhance the increase in LC3II induced by bosutinib, indicating that bosutinib inhibited autophagic flux in melanoma cells [216], which shows a similar effect with imatinib in treating HCC cells (34087223, 35182693). However, much remains to be explored in mechanistic studies of bosutinib, such as whether this drug induces autophagy in CML and whether bosutinib-induced autophagy plays a key role in bosutinib resistance.

#### 2.5.3. Dasatinib

Dasatinib, a second-generation tyrosine kinase inhibitor, is a highly effective drug for the treatment of Bcr-Abl-positive leukemia. Morita et al. demonstrated the presence of autophagosomes in the bone marrow, spleen, and brain of NOG mice injected with human leukemia cells (Bcr-Abl-positive) via tail vein after a single treatment with Dasatinib [217]. In addition, Xie et al. found that dasatinib induces myeloid differentiation in acute myeloid leukemia (AML) cells and concomitantly induces autophagy. The pharmacological autophagy inhibitors 3-MA, Wortmannin, LY294002, and CQ blocked dasatinib-induced AML cell differentiation, while the autophagy inducer rapamycin enhanced AML cell differentiation, indicating that autophagy enhances the dasatinib-induced differentiation. They also demonstrated that dasatinib enhanced all-trans-retinoic acid-induced differentiation capability through the initiation of autophagy [218]. Dasatinib has also been reported to have some proliferation inhibitory effects in many solid tumors, but the role of autophagy in this process is inconsistent. Hegedüs et al. reported that dasatinib increased the autophagic flux, as assessed by the degradation of the autophagy substrate p62/SQSTM1 and increased levels of LC3II, an increased number of GFP-LC3 puncta, and decreased readings of the luminescence of the HiBiT-LC3 reporter in different malignant pleural mesothelioma cells. Their data suggest that autophagy served as a cytoprotective mechanism following dasatinib treatments in malignant pleural mesothelioma cells [219]. Le et al. demonstrated dasatinib-induced autophagy by acridine orange staining, LC3 western blotting in vitro, and electron microscopy in xenograft tumor sections. shRNA knockdown of Beclin 1 expression reduced dasatinib-induced autophagy and growth inhibition, implying that dasatinib induces autophagic death in ovarian cancer cells [220]. However, reports on the mechanism of dasatinib-induced autophagy are very limited, and more studies are still needed.

#### 2.5.4. Nilotinib

Nilotinib, a second-generation TKI, is 30-fold more potent than imatinib in binding to BCR-ABL in treatment-resistant CML and 5 to 7-fold more potent than imatinib in imatinib-sensitive leukemia cells [221]. In addition to inactivating BCR-ABL, nilotinib inhibits kinases such as KIT, discoidindomain receptor (DDR), MAPK, ZAK, and PDGFR, but with less potency [222]. Due to its broad-spectrum kinase inhibitory activity, nilotinib can be further used for the treatment of other types of cancer, such as GIST, breast cancer, and melanoma [223]. In addition, Yu et al. found that nilotinib also showed inhibitory activity against hepatocellular carcinoma (HCC). Staining with acridine orange and LC3 revealed that nilotinib induced autophagy in a dose- and time-dependent manner in HCC cell lines, including PLC5, Huh-7, and Hep3B [224]. Beyond this finding, there are no studies on nilotinib-induced autophagy in tumor cells. However, a recent report might provide some guidance for future studies of nilotinib. Nilotinib is more effective in patients with chronic or accelerated stages of CML who are imatinib-resistant or imatinib-intolerant. However, according to clinical trial results, renal impairment was observed in approximately 17% of patients treated with nilotinib [225]. Persistent renal complications require a reduction in the nilotinib dose or discontinuation of treatment, which leads to uncontrolled cancer progression. Yan et al. found that nilotinib induced apoptosis by specifically reducing BCL2-like 1 (Bcl-XL) levels and nephrotoxicity in kidney cells. CQ intervenes with nilotinib-induced apoptosis and improves mitochondrial integrity, reactive oxygen species accumulation, and DNA damage by reversing the decreased Bcl-XL. The effect of intervention was dependent on the alleviation of nilotinib-induced reduction in ubiquitin-specific peptidase 13 (USP13) and not on autophagy inhibition [226]. This study suggests that CQ or HCQ could provide an intervention strategy for nilotinib’s nephrotoxicity, independent of autophagy inhibition. While the role that autophagy may play in the treatment of CML with nilotinib is currently unknown, this study provides a new way of thinking when conducting clinical study designs for the combination of CQ and nilotinib.

#### 2.5.5. Ponatinib

As a third-generation tyrosine kinase inhibitor, ponatinib eliminates BCR-ABL wild-type and mutant (BCR-ABL^T315I^) CML cells more effectively than first- and second-generation BCR-ABL tyrosine kinase protein inhibitors, thus reducing the evolution of resistance that may result from drug exclusion [227]. In addition, ponatinib has been shown to be more effective in inhibiting the growth of neuroblastoma cells [228]. Autophagy is often detected during ponatinib-induced tumor cell death. Mitchell et al. demonstrate that genetic or pharmacological inhibition (HCQ) of autophagy sensitizes ponatinib-resistant CML cells to death induced by mTOR inhibition in vitro and in vivo. Additionally, they show that catalytic mTOR inhibitors PI-103 and its derivative NVP-BEZ235 (which inhibit both mTORC1 and mTORC2 and have activity against all PI3K isoforms) induce autophagy and implied that mTOR inhibition could serve as an alternative therapeutic approach in TKI-resistant CML cells [229]. However, the role that autophagy plays in ponatinib-induced cell death in CML has not been further reported, and there is only one study available to us. Corallo et al. found that in neuroblastoma cells and wild-type zebrafish embryos, ponatinib induces the accumulation of autophagy vesicles. Inhibition of autophagic flux by CQ restores the cytotoxic potential of ponatinib, thus attributing a cytoprotective function to autophagy. In mice, the use of CQ as adjuvant therapy significantly improves the anti-tumor effects obtained by ponatinib, leading to an improved reduction of tumor masses [230], indicating that autophagic flux inhibition enhances the cytotoxicity of ponatinib.

### 2.6. MEK1/2

The ERK1/2 pathway is one of the most critical signaling pathways in the family of mitogen-activated protein kinases (MAPKs). While MEK1/2 is responsible for transmitting signals from various upstream kinases, it is the sole activator of downstream ERK1/2 and is therefore known as the “gatekeeper” of ERK1/2. Mutations in the RAS, RAF, and MEK1/2 genes lead to cancer, and in particular, RAS mutations and BRAF^V600^ mutations are very common in human cancers [231]. Studies have shown that MEK1/2 inhibitors are effective against tumors with both RAS and RAF mutations. A number of allosteric MEK inhibitors have been reported, most of which have a similar diarylamine scaffold [232]. Notably, the FDA-approved MEK inhibitors as well as the reported MEK degraders are based on this scaffold design. To date, four MEK inhibitors have been approved by the FDA, including trametinib for melanoma in 2013 [233], cobimetinib for BRAF-mutated advanced melanoma in 2015 [234], binimetinib for unresectable or metastatic melanoma with BRAFV^600E^ or BRAFV^600K^ mutations in 2018 [235], and selumetinib, approved in 2020 for type 1 neurofibromatosis [236].

Studies have shown that under nutrient-sufficient conditions, the proliferation of some KRAS-mutant cancer cell lines is not strongly affected, or is relatively little affected, by pharmacological inhibition of autophagy, RNAi-mediated acute ATG5/ATG7 knockdown, or CRISPR-mediated ATG7 knockdown [237,238]. However, the cytotoxicity of BRAF and CRAF siRNAs is enhanced when ATG7 is knocked down, suggesting that KRAS-driven metabolic alterations in cancer cells make them particularly dependent on the autophagy pathway as a survival mechanism when the RAF/MAPK pathway is acutely inhibited [238]. This implies that combining autophagy inhibitors with MEK inhibitors could prove to be an effective strategy for treating tumors with RAS mutations. Indeed, there are many preclinical studies that support this hypothesis. For example, Bryant et al. found that the autophagy inhibitor CQ and genetic or pharmacologic inhibition of specific autophagy regulators synergistically enhanced the ability of ERK inhibitors (SCH772984) to mediate antitumor activity in KRAS-driven pancreatic ductal adenocarcinoma (PDA) [239]. 

Of the FDA-approved MEK inhibitors, trametinib has been the most intensively studied. Kinsey et al. [240] showed that inhibition of MEK1/2 resulted in activation of the LKB1→AMPK→ULK1 signaling axis, which is a key regulator of autophagy. Furthermore, combined inhibition of MEK1/2 and autophagy produced synergistic antiproliferative effects on PDA cell lines in vitro and promoted tumor regression in mouse xenografts of PDA patients. The effects observed with trametinib in combination with CQ were not limited to pancreatic cancer; patient-derived xenografts (PDX) of other tumors, including NRAS-mutated melanoma and BRAF-mutated colorectal cancer, showed similar responses. Furthermore, a patient with PDA treated with the combination of trametinib and HCQ experienced a partial but significant disease response [240]. Similarly, Bhatt et al. found that the combination of HCQ and trametinib resulted in synergistic anti-proliferative activity in Kras^G12D/+^;Lkb^1−/−^(KL) lung cancer cells, but not in Kras^G12D/+^;p53^−/−^(KP) lung cancer cells. In vivo studies using tumor allografts, genetically engineered mouse models, and PDXs demonstrated the anti-tumor activity of the combination of HCQ and trametinib on KL but not KP tumors [241]. Co-mutations of LKB1 or TP53 with KRAS define distinct subsets of NSCLC that respond differently to standard cancer treatments. Taken together, the study of Bhatt suggests that LKB1 mutations could be explored as a predictive biomarker for precision lung cancer therapy using autophagy inhibitors [241]. Degan et al. showed that cotreatment of CQ and trametinib markedly slowed melanoma growth induced in Tyr-CreER.BrafCa.Ptenfl/fl mice. Of additional significance, the tissues treated with CQ and trametinib had significantly decreased numbers of CD4+ and CD8+ T-lymphocytes and F4/80+ macrophages [242]. This study suggested that a combination of HCQ and MEK inhibition could be a promising therapeutic strategy to specifically treat tumors bearing mutations of LKB1, KRAS, or BRAF. Not only CQ or HCQ but also other autophagy inhibitors have similar effects. Econazole, an antifungal compound that promotes the initiation of autophagy but hinders lysosomal biogenesis, acts synergistically with trametinib against PDA in vitro and in vivo [243]. However, there are examples in the literature when these autophagy inhibitors do not act in precisely the same manner. Truong et al. reported that treatment of mice bearing GNAQ/11-driven melanomas with trametinib plus HCQ resulted in inhibition of tumor growth and significantly prolonged survival. But interestingly, lysosomal- and autophagy-specific inhibition with Baf A1 was not sufficient to promote cytotoxicity in combination with trametinib [244]. 

The promising results of these preclinical trials prompted researchers to conduct a number of clinical studies combining CQ with trametinib. Silvis et al. found that the combination of trametinib plus CQ or HCQ demonstrated striking anti-tumor effects in preclinical models and in a patient (Patient 1). However, not all patients respond to the trametinib/HCQ regimen, and Patient 1 eventually developed resistant disease [245]. In patients with mPDAC who received the combination of trametinib and HCQ as third- or later-line therapy, it was found that the combination of trametinib and HCQ may not be an effective late-stage treatment for mPDAC [246]. Although the results of these clinical trials do not appear to be particularly encouraging, in a recent BAMM (BRAF Autophagy and MEK Inhibition in Melanoma) trial (NCT02257424), a four-center, phase I/II trial of dabrafenib, trametinib, and HCQ in BRAF inhibitor-naïve patients with advanced BRAF mutant melanoma, HCQ + dabrafenib+trametinib was well tolerated and produced a high response rate (RR) but did not meet criteria for success for the one-year progression-free survival (PFS) rate. In this difficult-to-treat population, the RR and PFS were encouraging. The BAMM regimen produced an 88% response rate in patients with elevated serum LDH. Nevertheless, the 85–88% response rate with this well-tolerated regimen is noteworthy and demonstrates the potential of autophagy inhibition for BRAF mutant cancers [208,247].

NSCLC displays activated MEK/ERK signaling due to the high frequency of K-Ras gene mutations and is therefore also a potential candidate for MEK-targeted therapies. Yao et al. found that binimetinib induced autophagy by inhibiting the PI3K/Akt pathway in NSCLC cells. Combined use of CQ with binimetinib synergistically inhibited NSCLC cell growth and enhanced apoptosis [248]. Similarly, Grasso et al. showed that autophagy induction by rapamycin increased cell survival, whereas pharmacology autophagy inhibition by bafilomycin A1, CQ, or 3-MA increased selumetinib-induced colorectal cancer cell death [249]. However, a recent clinical trial (NCT04735068) was disappointing in that the combination of binimetinib with HCQ in second- or later-line treatment of advanced KRAS-mutant NSCLC did not exhibit meaningful antitumor activity [250]. It is possible that simply combining an autophagy inhibitor with a MEK inhibitor is not sufficient to inhibit tumor progression. Jiang et al. [251] recently found that collateral inhibition of MEK (using cobimetinib) and autophagy (using mefloquine), but not either treatment alone, activates the STING/type I interferon pathway in tumor cells, which in turn activates paracrine tumor-associated macrophages toward an immunogenic M1-like phenotype. This switch is further augmented by CD40 agonism (aCD40). Triple therapy (cobimetinib + mefloquine + aCD40) achieved cytotoxic T-cell activation in an immunologically ‘cold’ mouse PDA model, leading to enhanced antitumor immunity [251]. This triple-combination therapy may prove to be a useful antitumor strategy. 

### 2.7. BTK Inhibitors

#### Ibrutinib

Ibrutinib is an irreversible and selective BTK inhibitor used to target human lymphoma, glioma, ovarian, breast, lung, and gastric cancers. Additionally, ibrutinib showed superior efficacy in targeting B-cell cancers such as mantle cell lymphoma and chronic lymphocytic leukemia [252]. Regarding autophagy, Sun et al. [253] showed that ibrutinib induced the autophagic machinery, as evidenced by LC II and Atg7 upregulation in the HS-4 skin cancer cell line. Combining ibrutinib with 3-MA increased apoptosis induction as compared with ibrutinib alone, highlighting the cytoprotective autophagy induced by ibrutinib.

In agreement with the Sun et al. [253] studies, Wang et al. [254] reported that ibrutinib induced cytoprotective autophagy in glioblastoma. Ibrutinib induced autophagic flux in LN229 and U87 cell lines by autophagosome accumulation, as visualized via TEM, LC3II, and ATG7 upregulation. Co-treatment with 3-MA potentiated the cytotoxicity of ibrutinib in LN229 and U87 cell lines, suggesting a cytoprotective role of the autophagic machinery. The latter observation was confirmed using ATG7-directed siRNA, where autophagy depletion enhanced the ibrutinib-mediated reduction in cell viability. Wang et al. [254] extended and confirmed these results in vivo using U87 cell xenograft mouse models, where the combination of ibrutinib and 3-MA was more effective than each therapy alone. Cytoprotective autophagy induction by ibrutinib in vivo was demonstrated by elevated LC3II expression.

### 2.8. TRK Inhibitors

#### 2.8.1. Entrectinib

Entrectinib is a potent new pan-TRK and ALK inhibitor used in multiple types of cancer, including NSCLC and colorectal cancer [255]. Limited data are available on the relationship between entrectinib and the autophagic machinery. Aveic et al. [256] studied the role of autophagy in entrectinib efficacy in neuroblastoma. Initially, they showed that entrectinib demonstrated a dose-dependent reduction in the viability of multiple neuroblastoma cell lines bearing various states of the ALK gene, specifically NB3^R1275Q^, NB1^amp^, IMR32^wt^, and SHSY5Y^F1174L^ cells. The antitumor activity of Entrectinib was confirmed using a clonogenic survival assay as well as RT-PCR, with a significant decrease in Ki-67 expression upon entrectinib treatment in NB1, NB3, and SH-SY5Y cells. Importantly, entrectinib induced autophagic flux, more obviously in SH-SY5Y^F1174L^ cells than other cell lines, as shown by GFP-LC3, LC3II upregulation, and p62/SQSTM1 degradation. Additionally, combining entrectinib with CQ resulted in a marked increase in cytotoxicity as compared with each drug alone, as evidenced by the TUNEL assay and flow cytometry for apoptosis, indicative of the cytoprotective role of the autophagic machinery induced by entrectinib in neuroblastoma.

#### 2.8.2. Larotrectinib

Larotrectinib is a potent and selective TRK inhibitor, approved for treating advanced TRK-positive solid tumors in both pediatric and adult patients [257]. As is the case with entrectinib, there are limited data in the scientific literature as to the role of autophagic flux in larotrectinib efficacy. Kong et al. [258] investigated larotrectinib activity in colorectal carcinoma using COLO205 and HCT116 colon cancer cell lines. Larotrectinib treatment reduced the viability of the two cell lines, as shown by a CCK-8 assay, in addition to suppressing the metastatic capacity of these cell lines, as evidenced by a transwell migration assay. Larotrectinib triggered the autophagic machinery in both cell lines, as shown by LC3 I/II conversion as well as p62/SQSTM1 degradation. To investigate the role of autophagy, larotrectinib in combination with CQ blocked larotrectinib-induced anti-migratory capacity as well as abrogated the larotrectinib-induced reduction in Ki-67, suggesting a cytotoxic role of autophagic machinery. Larotrectinib promoted AMPK upregulation with mTOR suppression, where siRNA-mediated knockdown of AMPK suppressed Larotrectinib-induced anti-migratory capacity in COLO205 and HCT116 colon cancer cell lines, with studies confirming that larotrectinib induced cytotoxic autophagy via the AMPK pathway. These findings were confirmed in nude mice injected subcutaneously with HCT116 cells, where larotrectinib treatment reduced tumor volumes and weight, inducing AMPK phosphorylation with suppression of mTOR, as well as causing LC3I/II conversion.

### 2.9. JAK Inhibitors

#### Ruxolitinib

Ruxolitinib, an FDA-approved selective inhibitor of JAK1/2, is approved for treatment of intermediate- and high-risk patients with primary myelofibrosis (PMF) [259]. The relation between ruxolitinib and autophagy was investigated by Neto et al. [260], where they showed that ruxolitinib treatment resulted in significant suppression of mTOR-related proteins as well as a significant consumption of LC3I/II and p62/SQSMT1 levels, indicating an active autophagic flux in SET-2 cells, a megakaryoblastic cell line [261]. These investigators studied the role of the autophagy machinery in this system using autophagy inhibitors, 3-MA, BAF A1, and CQ. Upon combining ruxolitinib with the different autophagy inhibitors, a significant increase in the apoptosis level was evidenced by annexin V/PI staining and caspase 3 cleavage as compared with ruxolitinib alone, suggesting a cytoprotective role of autophagy [260]. Similarly, Courdy et al. [262] showed cytoprotective autophagy in the cells harboring the JAK2^V617F^ mutation, including SET-2 cells and HEL cells, myeloproliferative neoplasms. Ruxolitinib treatment induced an active autophagic flux in these cell lines, as confirmed by LC3II accumulation and autophagosome aggregation. They then showed that autophagy inhibition, using CQ or SAR405 [263], significantly increased the cytotoxicity of ruxolitinib, confirming the cytoprotective role of the autophagic machinery [262].

### 2.10. PDGFR Inhibitors

Originally identified within platelets, platelet-derived growth factor (PDGF) serves as an α-granule component released in an autocrine manner following platelet activation. PDGF has been characterized as a pro-angiogenic factor, exerting crucial regulatory effects on physiological and pathological vascular networks [264]. PDGFs, along with their corresponding receptors (PDGFRα and PDGFRβ), are expressed in diverse malignant tumor cells and tissues, including but not limited to NSCLC, gastrointestinal stromal tumor (GIST), pancreatic cancer, breast cancer, ovarian carcinoma, hepatocellular carcinoma (HCC), and neuroendocrine tumors [265,266,267]. Substantial evidence indicates that PDGF actively contributes to tumor cell proliferation, angiogenesis, migration, and invasion, with a particular emphasis on malignant neoplasms [268]. PDGFR inhibitors can be categorized into two classes based on their binding characteristics with PDGFRα and/or PDGFRβ: specific inhibitors and non-specific inhibitors. Avapritinib is a selective tyrosine kinase inhibitor (TKI) that has received FDA approval for the treatment of metastatic or unresectable gastrointestinal stromal tumors harboring a platelet-derived growth factor receptor α (PDGFRA) exon 18 mutation [269]. These mutations are frequently encountered in individuals exhibiting resistance to conventional therapeutic approaches for gastrointestinal stromal tumors (GIST) [270]. Furthermore, avapritinib demonstrates relevance in cases of systemic mastocytosis characterized by the prevalent overexpression of KIT, a tyrosine kinase receptor [78,271]. Ripretinib, another PDGFR inhibitor, is a novel type II tyrosine switch control inhibitor designed to broadly inhibit activating and drug-resistant mutations in KIT and PDGFRA and has been approved for the treatment of TKI-refractory GIST [272]. Further research is necessary to identify a role for avapritinib- and ripretinib-induced autophagy in tumor cell survival.

## 3. Conclusions

Tyrosine kinase inhibitors (TKIs) have shown quite extensive clinical effectiveness in the clinical setting in treating various malignancies. The first approved TKI to be utilized in oncological settings was imatinib, followed by the approval of gefitinib, erlotinib, sorafenib, sunitinib, and dasatinib [273]. These agents have a different spectrum in targeting one to several tyrosine kinase proteins [273]. As is frequently the case with other anti-neoplastic agents, resistance development greatly limits the clinical efficacy of the TKIs. Autophagy is a cytoplasmic mechanism that serves to ensure cellular survival in response to various forms of endogenous and exogenous stress [10,31]. Autophagy has been extensively studied for its potential contribution to drug resistance [24,274]. The nature of the autophagy that is induced in response to various chemotherapeutic modalities may depend on the cell line/tumor model being utilized as well as the chemical nature of the therapeutic modality [10,29]. As summarized in Table 1, the majority of TKIs with different tyrosine kinase targets were able to induce autophagy, with the predominant form being cytoprotective, where autophagy inhibition increased the effectiveness of TKI therapy. These promising results were translated, in some cases, into clinical trials to investigate the possible targeting of autophagy to increase the effectiveness of TKIs in the clinic. However, there are a number of cases where the preclinical studies identified cytotoxic autophagy induced by the TKIs. Furthermore, there continues to be a need for the development of more specific autophagy inhibitors with a better side-effect profile than HCQ. Additionally, a non-invasive methodology for the assessment of autophagy promotion and inhibition in patients is needed in order to analyze and interpret the outcomes of clinical trials [30,275]. 

## Figures and Tables

**Table 1:** Different roles of autophagy induced in response to TKIs.

TKI Inhibitor	Class	Cell Lines/Tumor Type	Role of Autophagy	References
Afatinib	ERBB inhibitor	Head and neck squamous cell carcinoma (HNSCC) cells; FaDu, HN6, and CAL-27 cell lines; and in vivo using nude mice inoculated with H1975 cells	Cytoprotective	[45]
		lung adenocarcinoma with activating EGFR mutations using H1650 and H1975 cells	Cytoprotective	[46]
Lapatinib	ERBB inhibitor	HER2 breast cancer using parental BT-474 and AU-565 cells and the resistant cells BT-474LapR and AU-565LapR	Cytoprotective	[49]
		HER2 breast cancer; BT474 and AU565 cell lines	Cytotoxic	[50]
		AML-derived U937 and K562 cell line	Cytotoxic	[51]
		HCC cell lines, including Huh7, HepG2, and HA22T cells	Cytotoxic	[52]
		Esophageal carcinoma using HER2-positive, sensitive, and resistant OE19 cell lines	Cytoprotective	[53]
lapatinib and gefitinib		T24 and J82 human bladder cancer cells	Cytoprotective	[55]
Brigatinib	ALK inhibitor	ALK positive cells, H3122 and H2228 NSCLC cells, as well as ALK-negative cell lines, A549 (NSCLC), Hep3B (HCC), Du145 (brain), and HCT116 (lung) cells Colorectal cancer cell lines (DLD-1, HCT116, HT29, RKO, SW620): human colon mucosal epithelial cell line, NCM460 In vivo using nude mice subcutaneously inoculated with DLD-1 cells	Cytoprotective	[58]
Lorlatinib	ALK inhibitor	ALK-positive NSCLC cells, H3122, and H2228 cell lines In vivo using H3122 xenograft mouse model	Cytoprotective	[64]
Crizotinib	ALK inhibitor	Lung cell lines, including SPC-A1, A549, and H2228 cells In vivo using SPC-A1 xenograft mouse models	Cytoprotective	[68]
		Lung H3122-sensitive cells and H3122CR-1-resistant cell lines In vivo using mouse xenografts injected with H3122CR-1-resistant cells	Cytoprotective	[69]
		ALK-positive large cell lymphoma cell lines, Karpas-299 and SU-DHL-1 cells, and ALK-negative FEBD cells In vivo using Karpas-299 xenograft mouse tumor models	Cytoprotective	[70]
		MET overexpressed, SNU-5, and MKN45 cells	Cytotoxic	[71]
Ibrutinib	BTK inhibitor	HS-4 skin cancer cell line	Cytoprotective	[253]
		LN229 and U87 glioblastoma cell lines In vivo using U87 cells xenograft mice models	Cytoprotective	[254]
Entrectinib	TRK inhibitor	Neuroblastoma cell lines bearing various statuses of ALK gene: NB3^R1275Q^, NB1^amp^, IMR32^wt^, and SHSY5Y^F1174L^ cells	Cytoprotective	[256]
Larotrectinib	TRK inhibitor	Colorectal carcinoma using COLO205 and HCT116 colon cancer cell lines In vivo using nude mice injected subcutaneously with HCT116 cell line	Cytotoxic	[258]
Ruxotinib	JAK inhibitor	Megakaryoblastic cell line, SET2 cells	Cytoprotective	[260]
		Myeloproliferative neoplasms models, HEL, and SET-2 cells	Cytoprotective	[262]
Dacomitinib	2^nd^-generation EGFR inhibitor	NSCLC cell lines (NCI-H1975, NCI-H1650, HCC827, A549, and NCI-H1299)	Cytoprotective	[121]
Erlotinib	1^st^-generation EGFR inhibitor	Lung cancer cell lines (A549, NCI-H1299, NCI-H292, NCI-H1650, and SK-MES-1)	Cytoprotective	[74]
		NSCLC cell lines (A549, H522, H1975, and PC9)	Cytotoxic	[101]
Gefitinib	1^st^-generation EGFR inhibitor	Glioblastoma cell lines (T98G, LN229, and U87MG)	Cytoprotective	[79]
		NSCLC cell lines (PC-9, A549, and H226) Leukemia cell lines (HL-60, K562, *Chop*^−/−^MEF and *Chop*^+/+^MEF)	Cytoprotective	[76]
		Sinus-derived squamous cell carcinoma cell lines (UM-SCC1 and PCI-15B) Oral squamous cell carcinoma cell lines (MDA-686LN) T-24 transitional cell carcinoma cell lines (Hela-R29 and Hela-R30)	Cytotoxic	[89]
		Lung cancer cell lines (HCC827 *EGFR* 19del, H1975 *EGFR* L858R, and T790M mutations)	Cytoprotective	[82]
Mobocertinib	EGFR inhibitor	NA	NA	NA
Osimertinib	3^rd^-generation EGFR inhibitor	NSCLC cell lines (PC-9GR and H1975)	Cytoprotective	[109]
		Lung cancer cell lines (PC-9, PC-9GR, and H1957)	Cytoprotective	[41]
		Cancer cell lines (DLD-1, HT29, HCT116, SW620, LoVo, RKO, and SW480)	Cytoprotective	[41]
Avapritinib	PDFGFR inhibitor	NA	NA	NA
Ripretinib	PDGFR KIT inhibitor	NA	NA	NA
Axitinib	2^nd^-generation VEGFR inhibitor	NA	NA	NA
Vandetanib	2^nd^-generation VEGFR inhibitor	NSCLC cell lines (Calu-6)	Cytoprotective	[134]
		Glioblastoma cell lines (U251 and U87MG) Mouse xenograft tumor model	Cytoprotective	[136]
		Glioma cell lines (C6) C6 tumor intracranial-bearing mice	Cytoprotective	[137]
Tivozanib	2^nd^-generation VEGFR inhibitor	NA	NA	NA
Sunitinib	2^nd^-generation VEGFR inhibitor	Medullary thyroid cancer cell lines (MTC, TT)	Cytotoxic	[167]
		Rat Pheochromocytoma cells (PC12)	Cytoprotective	[151]
		Human breast (MCF-7, T-47D), cervical (Hela), colorectal (Caco-2, HCT116), hepatocellular (HepG2), laryngeal (HEp-2) and prostate (PC3) cell lines. Ehrlich ascites carcinoma Swiss albino mouse models	Cytoprotective	[153]
		Pancreatic neuroendocrine cell lines (BON1 cell line)	Cytoprotective	[152]
Sorafenib	2^nd^-generation VEGFR inhibitor	Medullary thyroid cancer cell lines (MTC and TT)	Cytotoxic	[167]
		Renal cell carcinoma cell lines (786-0 and A489)	Cytoprotective	[162]
		Hepatocellular Carcinoma cell lines (PLC5 and SK-Hep1) Male NCr athymic nude mice	Cytotoxic	[159]
		Hepatocellular Carcinoma cell lines (Bel-7402 and HepG2)	Cytoprotective	[164]
Regorafenib	2^nd^-generation VEGFR inhibitor	Glioblastoma multiforme cell lines BALB/c nude mice and NOD/SCID mice Zebrafish xenograft model	Cytotoxic	[173]
		Hepatocellular carcinoma cell lines (HepG2 and Huh7) Sorafenib-resistant HepG2 and Huh7 cells	Cytotoxic	[174]
Pazopanib	2^nd^-generation VEGFR inhibitor	Bladder cancer cell lines (p53 mutant, 5637, and J82)	Cytoprotective	[154]
Lenvatinib	2^nd^ generation VEGFR inhibitor	Papillary thyroid cancer cell lines (K1 and BCPAP) Lenvatinib treated nude mice	Cytoprotective	[179]
		Gallbladder cancer cell lines (GBC-SD and NOZ) Lenvatinib treated immunodeficient BALB/c nude mice	Cytoprotective	[180]
Cabozantinib	2^nd^-generation VEGFR inhibitor	Renal cancer cell lines (786-0, ACHN, Caki-1, and Caki-2)	Cytotoxic	[145]
		Colorectal carcinoma cell lines (HCT116 and HT29)	Cytoprotective	[143]
Imatinib	1^st^-generation BCR-ABL inhibitor	Gastrointestinal stromal tumor cells (GIST-T1 and GIST882 cells)	Cytoprotective	[207]
		Gastrointestinal stromal tumor cells (GFRA1-positive GIST-882 cells)	Cytoprotective	[208]
		Mouse xenograft model derived from gastrointestinal stromal tumor cells	Cytoprotective	[209]
		Imatinib-resistant GIST cells	Cytoprotective	[210]
Bosutinib	2^nd^-generation BCR-ABL inhibitor	Melanoma cells	NA	[216]
Dasatinib	2^nd^-generation BCR-ABL inhibitor	Malignant pleural mesothelioma cells (SPC111and and SPC111 cell lines)	Cytoprotective	[219]
		Ovarian cancer cells (SKOv3 and HEY cells)	Cytotoxic	[220]
Nilotinib	2^nd^-generation BCR-ABL inhibitor	NA	NA	NA
Ponatinib	3^rd^-generation BCR-ABL inhibitor	Neuroblastoma (SH-SY5Y cells), wild-type zebrafish embryos, and IMR-32-bearing mice	Cytoprotective	[230]
Trametinib	MEK inhibitor	Lung cancer cells	NA	[241]
		Melanoma in TyrCreER.BrafCa.Ptenfl/fl mice	Cytoprotective	[242]
		Mice bearing GNAQ/11-driven melanomas	Cytoprotective (plus HCQ, not BAF A1)	[244]
Binimetinib	MEK inhibitor	Non-small cell lung cancer (A549 cells)	Cytoprotective	[248]
Selumetinib	MEK inhibitor	Colorectal cancer cells (SW480 and HT29)	Cytoprotective	[249]

NA: not applicable.

## Data Availability

No new data were created or analyzed in this study. Data sharing is not applicable to this article.
